# Lignin‐Containing Nanocellulose Mediated Interlayer Modulation Unlocks Stable and Redispersible MXene

**DOI:** 10.1002/advs.202508665

**Published:** 2025-10-03

**Authors:** Shuyang He, Zhen Yu, Shan Li, Shijie Lei, Lin Zhu, Ke Zhao, Fangxia Yang, Ningning Cao, Yuyan Liu, Zhimin Fan

**Affiliations:** ^1^ Shaanxi Provincial Key Laboratory of Economic Plant Resources Development and Utilization College of Forestry Northwest A&F University Yangling 712100 China; ^2^ School of Environmental Science and Engineering Tianjin University Tianjin 300072 China; ^3^ Air Defense and Antimissile School Air Force Engineering University Xi'an 710100 China; ^4^ School of Chemistry and Chemical Engineering Beijing Institute of Technology Beijing 102488 China; ^5^ College of Engineering and Applied Sciences Nanjing University Nanjing 210093 China; ^6^ State Key Laboratory of Space Power‐Sources School of Chemistry and Chemical Engineering Harbin Institute of Technology Harbin 150001 China

**Keywords:** long‐term redispersion, scalable, semisolid, structurally fixable, Ti_3_C_2_T_x_ MXene

## Abstract

Titanium carbide (Ti_3_C_2_T*_x_*) MXene combines exceptional conductivity, mechanical robustness, and multifunctionality, positioning it as a promising material for diverse applications. However, its industrial deployment remains hampered by the inability to precisely control redispersibility and oxidative stability. Herein, an interlayer chemical modulation strategy is reported, mediated by amphiphilic lignin‐containing nanocellulose (LNC). Competitive interactions between the hydrophilic and hydrophobic segments of LNC within MXene interlayers enable precise tuning of spacing and interfacial chemistry. This approach allows rapid, industrial‐scale spray drying to produce semi‐solid MXene with long‐term reversible redispersibility and outstanding oxidative stability. The resulting material can be fully redispersed into monolayer MXene even after 180 days of storage while maintaining high conductivity (≈7000 S cm^−1^). Moreover, by adjusting the post‐drying time‐dependent window, MXene powders can be programmably switched from a dynamically reversible to a permanently fixed structure, broadening their utility across multiple domains. This approach is expected to resolve the long‐standing industry bottlenecks of MXene, including its susceptibility to oxidation, high transportation costs, and challenges in reprocessing, thereby opening a new path for the rapid transition of MXene from laboratory to commercial application.

## Graphical abstract

Amphiphilic lignin‐containing nanocellulose (LNC) establishes competitive interactions between MXene nanosheets, enabling precise modulation of interlayer spacing and interfacial chemistry. This strategy allows rapid fabrication of semi‐solid MXene via industrial‐scale spray drying, achieving long‐term reversible redispersibility and exceptional oxidative stability, while providing theoretical and technological foundations to overcome critical bottlenecks in MXene's large‐scale production.

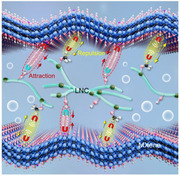

## 1 Introduction

Over the past fifteen years, 2D transition metal carbides, nitrides, and carbonitrides (collectively termed MXenes) have expanded into a diverse family of 2D materials. Their intrinsic hydrophilicity, metallic conductivity, and mechanical robustness have positioned MXenes as promising candidates for applications ranging from energy storage to electronics and biomedicine.^[^ ^1^, ^2^, ^3^, ^4^, ^5^, ^6^ ^]^ Among them, titanium carbide (Ti_3_C_2_T*_x_*, where T*_x_* denotes surface terminations such as ─OH, ─O, and ─F) MXene stands out for its balanced performance and scalable synthesis using established chemical‐engineering routes,^[^ ^7^, ^8^, ^9^ ^]^ offering a realistic prospect of industrial production. However, freshly prepared MXene typically exists as dilute aqueous dispersions that are vulnerable to oxidative degradation in the presence of water and oxygen,^[^ ^10^ ^]^ leading to property loss and high storage and transport costs. The intrinsically low solid content of these dispersions further hampers downstream processing, severely constraining the industrial translation of MXene.

The synthesis of MXene primarily relies on wet‐chemical etching, which imparts strong hydrophilicity and excellent processability while retaining the high conductivity of the parent MAX phase.^[^ ^11^ ^]^ As a result, water molecules are intimately involved at every stage, from etching and exfoliation to device assembly. Water not only serves as the etching medium but also governs the self‐assembly pathways and structural characteristics of MXene nanosheets. However, hydrolysis‐driven degradation is a key factor underlying MXene aging, particularly in dispersions where water accelerates oxidative degradation. As the proverb aptly states, “water can carry a boat, but it can also overturn it,” underscoring the dual role of water throughout the MXene lifecycle. Previous studies have shown that converting dispersions into fully solid‐state MXene films preserves functional stability for up to a decade,^[^ ^12^ ^]^ and that removing interlayer‐bound water greatly improves thermal endurance.^[^ ^13^ ^]^ These findings indicate that moisture isolation is crucial for sustaining MXene's long‐term structural and functional integrity. However, once MXene nanosheets are assembled into macroscopic solids such as films, fibers or aerogels, strong hydrogen bonding and van der Waals interactions render the material irreversibly fixed, eliminating redispersibility. This loss of reversibility poses a fundamental barrier to industrial translation, as scalable processing process from dispersions to devices critically depends on efficient redispersion. Without addressing this bottleneck, the practical versatility of MXene will remain severely constrained.

To address the intertwined challenges of redispersibility and oxidative stability, our team introduced the concept of “semi‐solid MXene” in 2020.^[^ ^14^ ^]^ By concentrating dilute dispersions into a semi‐solid state, we markedly reduced water‐driven oxidative degradation of MXene nanosheets, while the presence of residual water channels preserved long‐term reversible redispersion. Subsequent studies have also reported semi‐solid MXene formulations,^[^ ^15^ ^]^ demonstrating retention of dispersibility and performance stability for several months. Nevertheless, these materials still suffer from high water content, which poses oxidation risks, and their stability is limited to storage under low‐temperature conditions. In contrast, fully solid‐state MXene shows excellent resistance to oxidation but completely loses redispersibility. Semi‐solid MXene, therefore, represents a compromise between dispersions and solid‐state materials. Moreover, current preparation routes are time‐intensive (typically requiring hours to days), rendering them unsuitable for industrial‐scale production. Given MXene's game‐changing potential across critical technologies, once large‐scale dispersion production is achieved, balancing semi‐solid and fully solid states and realizing a rapid, controllable phase transition emerges as a central scientific and technological challenge for the field.

Here, we present an interlayer modulation strategy in which amphiphilic lignin‐containing nanocellulose (LNC) acts as a chemical mediator for MXene assembly. Coupled with an industrially viable spray‐drying process, this approach enables precise fixation of MXene dispersions in both semi‐solid and fully solid states. Competitive interactions between the hydrophilic and hydrophobic moieties of LNC within the MXene interlayers generate a defined time‐dependent window, during which fully solid MXene powders retain reversible redispersibility and can be rapidly converted into semi‐solid form. Beyond this window, LNC stabilizes the interlayer structure and macroscopic morphology, allowing direct use in applications such as electromagnetic absorption and electrochemical energy storage. This strategy provides both mechanistic insight and technological feasibility for overcoming long‐standing barriers in large‐scale MXene production, and offers a practical route toward its commercial realization.

## 2 Results and Discussion

**Figure 1:**
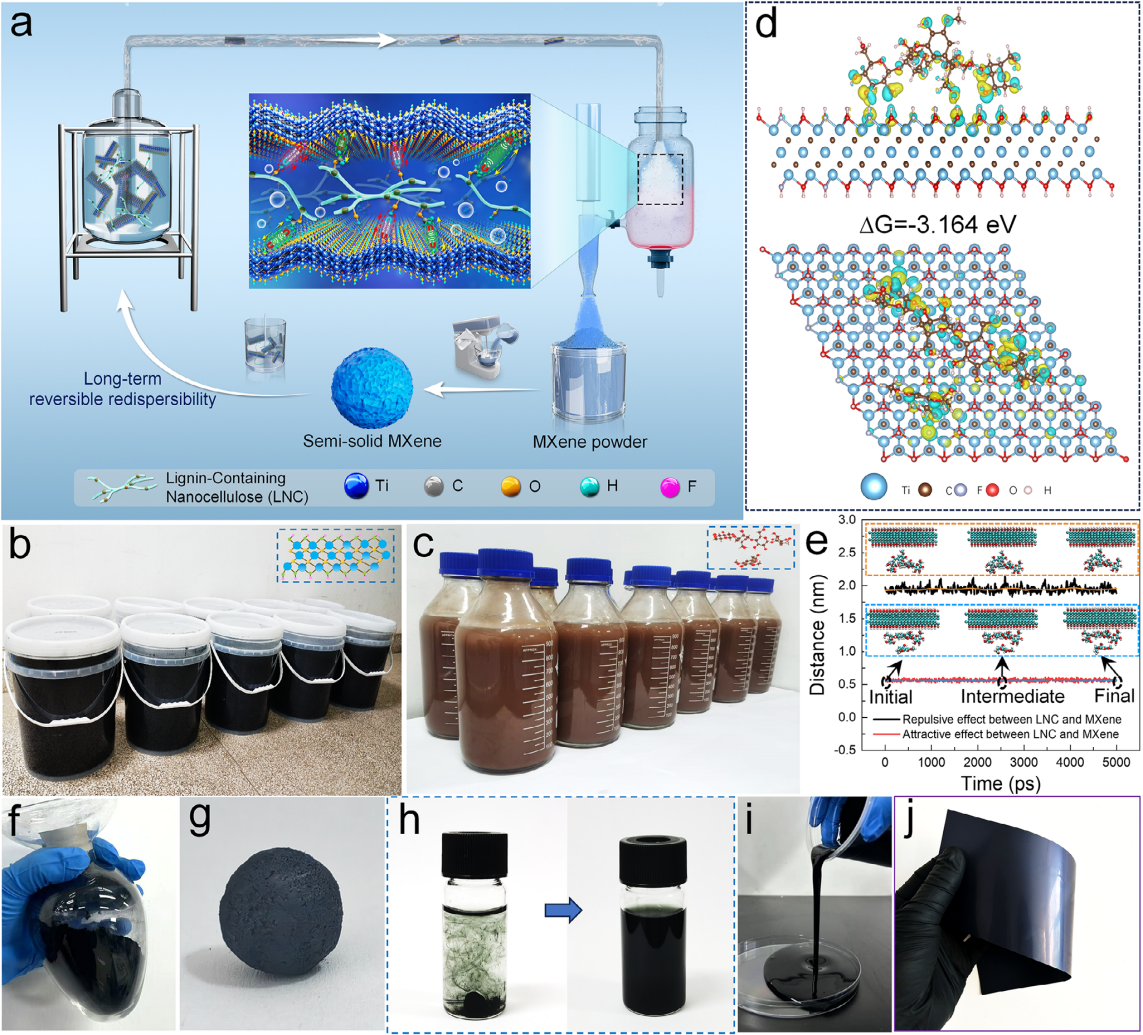
a) Schematic illustrating the rapid preparation of S‐MXene with reversible redispersibility via LNC‐mediated regulation. Photographs of b) MXene dispersion and c) LNC dispersion prepared in a single batch. d) Density functional theory calculations between LNC and MXene layers. e) Time‐dependent curve of the shortest distance between the hydrophobic and hydrophilic hydrogen atoms on the LNC chain segments and the hydrogen atoms on the terminal MXene surface. f) Photograph of the powder obtained by spray‐drying the MXene dispersion containing 1 wt.% LNC. g) Photograph of S‐MXene. h) Demonstration of the redispersibility of S‐MXene. Photographs of i) the ink after the transformation of S‐MXene and j) the film prepared by blade coating.

Figure 1a schematically illustrates the process of lignin‐containing nanocellulose (LNC)‐mediated interlayer modulation of MXene and the rapid preparation of semi‐solid MXene with reversible redispersibility via spray drying. The monolayer MXene (Ti_3_C_2_T_x_) dispersion (Figure 1b) was first synthesized using the LiF/HCl etching route (Figure S1, Supporting Information) combined with our high‐temperature ultrasonic exfoliation method.^[^ ^9^ ^]^ The diluted dispersion exhibited the characteristic light‐green color of Ti_3_C_2_T_x_ and a pronounced Tyndall effect (Figure S2, Supporting Information). The nanosheets displayed clean, smooth surfaces (Figure S3a,b, Supporting Information) with a thickness of approximately ≈1.4 nm (Figure S3c, Supporting Information), distinct from both the MAX precursor (Figure S4a, Supporting Information) and the multilayer MXene obtained after etching (Figure S4b, Supporting Information). Film assembled by vacuum exhibited a light purple appearance (Figure S5a, Supporting Information), excellent flexibility (Figure S5b, Supporting Information) and foldability (Figure S5c, Supporting Information), and a conductivity up to 7340 S cm−^1^, confirming the high quality and monolayer nature of the MXene.^[^ ^16^ ^]^ LNC was prepared by a green, low‐cost, and scalable ternary deep eutectic solvent (TDES) pretreatment combined with microfluidic mechanical fibrillation ^[^ ^17^, ^18^ ^]^ (Figure 1c). The resulting chain segments contained both hydrophilic and hydrophobic groups, imparting distinct amphiphilicity. Density functional theory (DFT) calculations revealed strong adsorption of LNC on MXene surfaces, with negative Gibbs free energy (Figure 1d). Molecular dynamics simulations further indicated that the shortest distance between hydrophilic groups of LNC and surface hydrogens on MXene was notably smaller than that of the hydrophobic groups (Figure 1e), reflecting preferential attraction of hydrophilic groups and repulsion of hydrophobic moieties. This dual interaction enables stable anchoring of LNC while balancing interlayer spacing, a mechanism elaborated below. Guided by these insights, industrial‐scale spray drying was employed to transform dilute dispersions containing 1 wt% LNC (denoted as P‐MXene) into powdery MXene (Figure 1f). The powders exhibited excellent redispersibility,; upon adding a small amount of water and simple kneading, they rapidly formed a semi‐solid MXene dough (S‐MXene) with a solid content of up to 45 wt.% (Figure 1g). The internal structures of S‐MXene was were relatively dense and disordered (Figure S6, Supporting Information), and the entire process from dispersion to semi‐solidification was completed within minutes, compared with hours or days required by conventional methods. Importantly, S‐MXene could be fully redispersed to its original monolayer state (Figure 1h; Figure S7, Supporting Information). Moreover, it could be readily processed into a conductive ink with excellent rheological behavior (Figure 1i) and further fabricated into a large‐area, self‐standing film (Figure **1**j).

**Figure** **1**a schematically illustrates the process of lignin‐containing nanocellulose (LNC)‐mediated interlayer modulation of MXene and the rapid preparation of semi‐solid MXene with reversible redispersibility via spray drying. The monolayer MXene (Ti_3_C_2_T*_x_*) dispersion (Figure 1b) was first synthesized using the LiF/HCl etching route (Figure S1, Supporting Information) combined with our high‐temperature ultrasonic exfoliation method.^[^ ^9^ ^]^ The diluted dispersion exhibited the characteristic light‐green color of Ti_3_C_2_T*_x_* and a pronounced Tyndall effect (Figure S2, Supporting Information). The nanosheets displayed clean, smooth surfaces (Figure S3a,b, Supporting Information) with a thickness of ≈1.4 nm (Figure S3c, Supporting Information), distinct from both the MAX precursor (Figure S4a, Supporting Information) and the multilayer MXene obtained after etching (Figure S4b, Supporting Information). Film assembled by vacuum exhibited a light purple appearance (Figure S5a, Supporting Information), excellent flexibility (Figure S5b, Supporting Information) and foldability (Figure S5c, Supporting Information), and a conductivity up to 7340 S cm^−1^, confirming the high quality and monolayer nature of the MXene.^[^ ^16^ ^]^ LNC was prepared by a green, low‐cost, and scalable ternary deep eutectic solvent (TDES) pretreatment combined with microfluidic mechanical fibrillation^[^ ^17^, ^18^ ^]^ (Figure 1c). The resulting chain segments contained both hydrophilic and hydrophobic groups, imparting distinct amphiphilicity. Density functional theory (DFT) calculations revealed strong adsorption of LNC on MXene surfaces, with negative Gibbs free energy (Figure 1d). Molecular dynamics simulations further indicated that the shortest distance between hydrophilic groups of LNC and surface hydrogens on MXene was notably smaller than that of the hydrophobic groups (Figure 1e), reflecting preferential attraction of hydrophilic groups and repulsion of hydrophobic moieties. This dual interaction enables stable anchoring of LNC while balancing interlayer spacing, a mechanism elaborated below. Guided by these insights, industrial‐scale spray drying was employed to transform dilute dispersions containing 1 wt% LNC (denoted as P‐MXene) into powdery MXene (Figure 1f). The powders exhibited excellent redispersibility; upon adding a small amount of water and simple kneading, they rapidly formed a semi‐solid MXene dough (S‐MXene) with a solid content of up to 45 wt.% (Figure 1g). The internal structures of S‐MXene were relatively dense and disordered (Figure S6, Supporting Information), and the entire process from dispersion to semi‐solidification was completed within minutes, compared with hours or days required by conventional methods. Importantly, S‐MXene could be fully redispersed to its original monolayer state (Figure 1h; Figure S7, Supporting Information). Moreover, it could be readily processed into a conductive ink with excellent rheological behavior (Figure 1i) and further fabricated into a large‐area, self‐standing film (Figure 1j).

**Figure 2:**
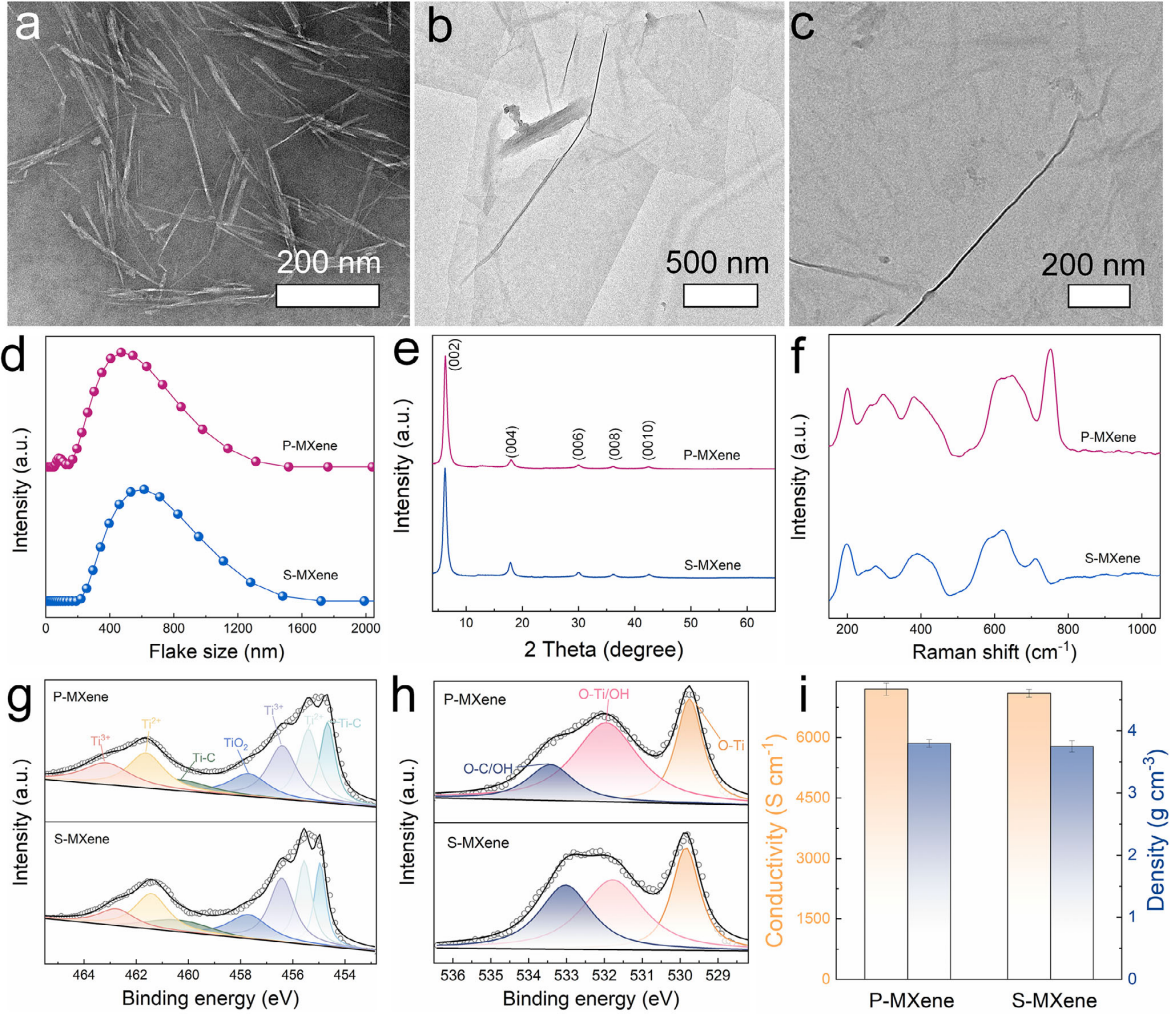
TEM images of a) LNC, b) S‐MXene after redispersion, and c) P‐MXene. d) Dynamic light scattering, e) XRD, and f) Raman of S‐MXene and P‐MXene. High‐resolution XPS spectra of g) Ti 2p and h) O 1s for P‐MXene and S‐MXene. i) Electrical conductivity and density of P‐MXene and S‐MXene (*n* = 5; error bars indicate the standard deviation).

To elucidate the reversible redispersibility of S‐MXene and the dual modulation effect of LNC on its nanosheets, we systematically examined its microstructure, composition, and physical properties. LNC disperses stably in water, showing no phase separation even after 30 days (Figure S8, Supporting Information), a stability attributed to its abundant hydrophilic ─OH groups. Transmission electron microscopy (TEM) (**Figure** **2**a) and atomic force microscopy (AFM) (Figure S9, Supporting Information) revealed that LNC adopts an ultrafine 1D tubular morphology. Importantly, the preparation of LNC is simple, economical, and readily scalable, with a single batch yielding ≈20 L of 2.5 wt.% dispersion under laboratory conditions (Figure 1c). Figure 2b presents a TEM image of redispersed S‐MXene, where nanosheets remain well‐dispersed, free of obvious oxidative particles, and decorated with fibrous LNC, closely resembling the morphology of P‐MXene (Figure 2c). Dynamic light scattering (DLS) analysis (Figure 2d) indicates that the size distribution of S‐MXene is nearly identical to that of P‐MXene. Furthermore, X‐ray diffraction (XRD) (Figure 2e), Raman spectroscopy (Figure 2f), and X‐ray photoelectron spectroscopy (XPS) (Figure 2g,h), along with conductivity and density measurements (Figure 2i), demonstrated that the physicochemical properties of redispersed S‐MXene are essentially indistinguishable from those of P‐MXene. Collectively, these results provide compelling evidence that semi‐solid MXene exhibits excellent reversible redispersibility, fully restoring to its initial state and thereby establishing a robust foundation for efficient MXene recycling.

**Figure 3:**
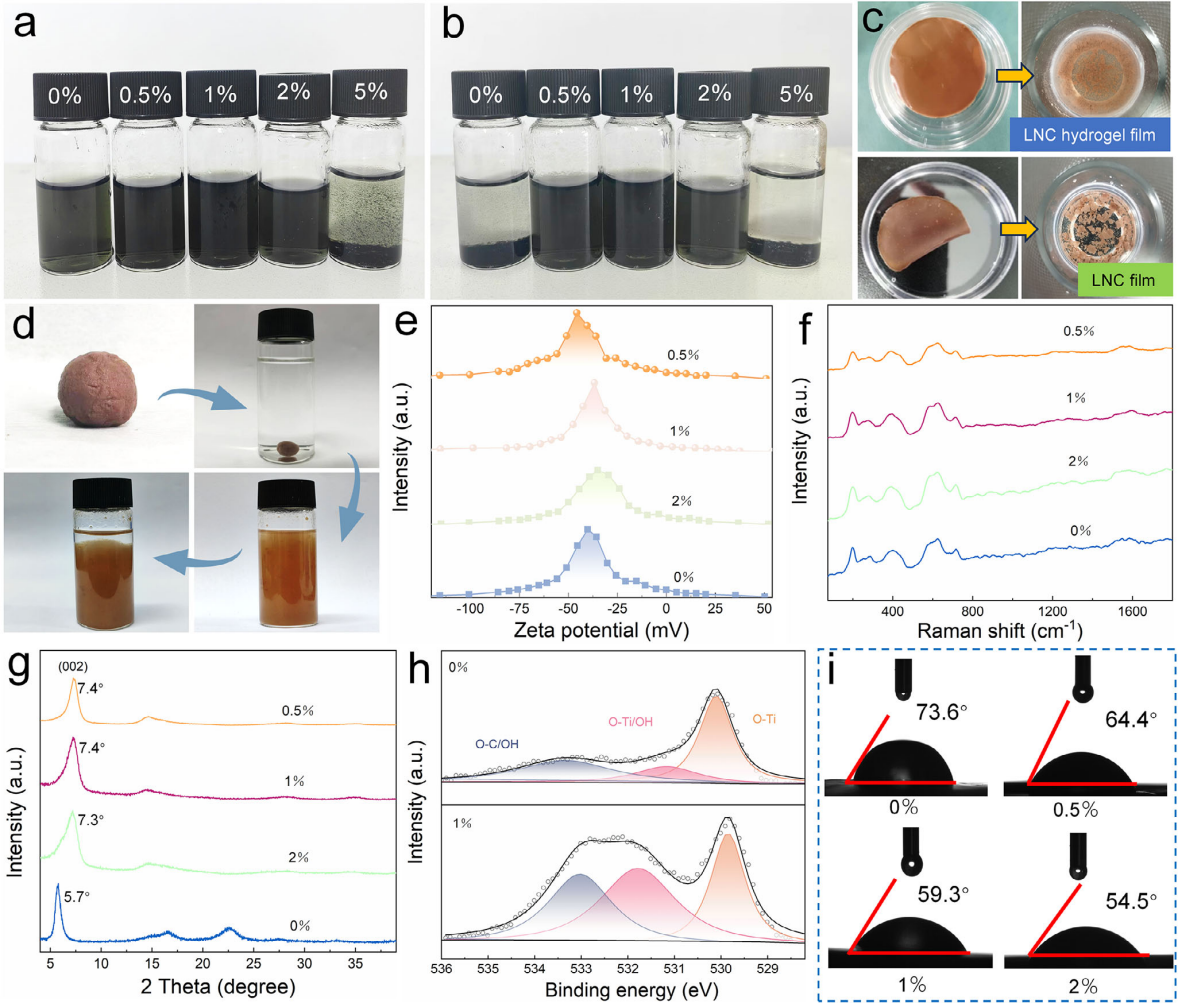
a) Powders obtained by spray‐drying MXene dispersions with different LNC contents after redispersion and b) after 5 days of storage. c) Redispersion of LNC hydrogel film versus dried LNC film. d) Redispersion of semi‐solid LNC dough. e) Zeta potential, f) Raman, and g) XRD spectra of redispersed semi‐solid MXene with different LNC contents. h) High‐resolution O1s XPS spectra of redispersed semi‐solid MXene with 0% and 1% LNC. i) Water contact angles of redispersed semi‐solid MXene with different LNC contents.

The LNC content plays a decisive role in both the spray‐drying behavior of MXene dispersions and the redispersibility of the resulting semi‐solid MXene. As shown in **Figure** **3**a, the pure MXene dispersion, when spray‐dried without LNC, yielded powders with poor redispersibility, undergoing complete sedimentation within 5 days (Figure 3b). Spray‐drying the pure LNC dispersion performed even worse, exhibiting noticeable sedimentation within minutes (Figure S10, Supporting Information). Similarly, at high LNC loading (5 wt.%), MXene powders largely precipitated (Figure 3b). By contrast, when the LNC content was maintained at 0.5, 1, and 2 wt%, the resulting powders exhibited excellent redispersibility in water, remaining stable after 5 days (Figure 3b). This behavior is attributed to excessive 1D LNC chains at high loadings, which undergo entanglement and crystallization,^[^ ^19^, ^20^ ^]^ thereby hindering redispersion. Supporting evidence comes from the poor redispersibility of high‐solid‐content LNC hydrogel film, dried LNC film (Figure 3c), and semi‐solid LNC dough (Figure 3d), where even slight increases in dispersion concentration (Figure S11, Supporting Information) markedly reduced redispersion capability. We next focused on semi‐solid MXene samples containing 0.5–2 wt.% LNC, all of which demonstrated robust redispersibility. As shown in Figure 3e, the Zeta potential of these redispersed systems remained stable at ≈−40 mV, comparable to fresh MXene dispersion, confirming preserved colloidal stability. Raman spectra of redispersed MXene films showed no significant changes relative to pristine samples (Figure 3f). Notably, the addition of even a trace amount of LNC induced a distinct shift of the (002) peak to a higher angle (Figure 3g), from 5.7° (d‐spacing 1.55 nm) to ≈7.4° (d‐spacing 1.19 nm), indicating nanosheet densification. This contrasts sharply with the interlayer expansion typically induced by conventional fillers, highlighting a unique compaction effect of LNC that preserves MXene's intrinsic performance. Notably, films reassembled from redispersed S‐MXene exhibited conductivities of 6800–7050 S cm^−1^ (Figure S12a, Supporting Information), nearly identical to that of pure MXene film (7340 S cm^−1^), while their tensile strength increased markedly to 11.3–14.7 MPa compared with 9.6 MPa for pure MXene film (Figure S12b, Supporting Information). Further evidence comes from XPS O1s spectra (Figure 3h), which show increased peak area ratios for O‐Ti (528.8 eV), O‐Ti/OH (530.7 eV), and O─C/OH (532.0 eV) in the S‐MXene film. The higher O─C/OH content suggests improved water adsorption, corroborated by progressively reduced water contact angles (Figure 3i). Collectively, these results demonstrate that introducing a small amount of LNC not only imparts excellent reversible redispersibility to spray‐dried MXene but also stabilizes its transition to a semi‐solid state, an outcome unattainable with pure MXene.

**Figure 4:**
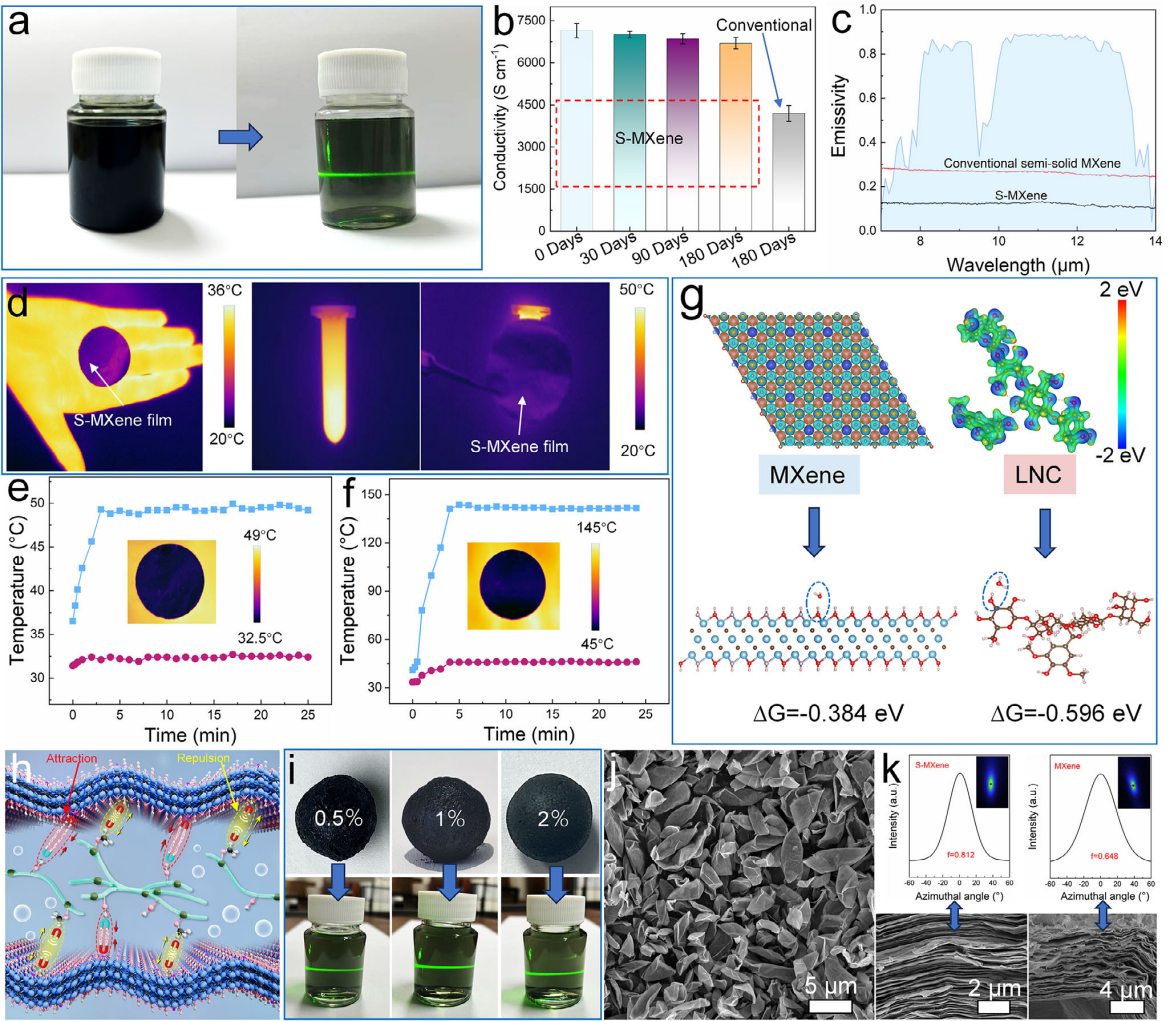
a) Redispersibility of S‐MXene sealed and stored at 5 °C for 180 days. b) Comparison of electrical conductivity of S‐MXene and conventional semi‐solid MXene after long‐term storage (*n* = 5; error bars indicate the standard deviation). c) Comparison of infrared emissivity of S‐MXene and conventional semi‐solid MXene after 180 days of storage. d) Infrared thermal images of the film prepared from S‐MXene after long‐term storage were placed on the palm and on the surface of a centrifuge tube filled with hot water. Change in surface radiative temperature over time of the film prepared from S‐MXene after long‐term storage on a hot plate at e) 50 °C and f) 145 °C. g) Comparison of the adsorption strength between the MXene terminal surface and water molecules, and between the LNC chain segments and water molecules. h) Mechanism of LNC‐mediated chemical regulation between the MXene layer. i) Demonstration of the redispersibility of semi‐solid MXene after transformation during the time‐dependent effect of MXene powders obtained by spray‐drying dispersions containing different LNC (0.5, 1, 2 wt.%). j) SEM image of the powder obtained by spray‐drying MXene dispersion containing 1 wt.% LNC. k) WAXS spectra of Cu‐Kα X‐ray beam incident parallel to the S‐MXene and MXene films layer plane and cross‐section.

The ability of semi‐solid MXene to sustain long‐term reversible redispersion while preserving its physicochemical properties is essential for industrial deployment. To systematically evaluate stability, S‐MXene was sealed at 5 °C for 180 days and subsequently tested. As shown in **Figure** **4**a, the stored sample could still be fully redispersed, yielding a light‐green dispersion devoid of visible particulates. TEM image confirmed that the nanosheet surfaces remained clean without detectable TiO_2_ particles (Figure S13, Supporting Information). This is consistent with the XPS Ti 2p spectra (Figure S14, Supporting Information), which showed no evident changes and no TiO_2_ characteristic peaks compared with the fresh sample (Figure 2g). By contrast, semi‐solid MXene prepared using our previous method^[^ ^14^ ^]^ exhibited a marked decline in redispersibility under identical conditions (Figure S15a, Supporting Information), with clear evidence of internal oxidation (Figure S15b, Supporting Information). XRD patterns of S‐MXene after 180 days (Figure S16, Supporting Information) likewise showed negligible variation relative to the fresh sample (Figure 2e). Since conductivity and infrared emissivity are key metrics of MXene quality, we further examined these parameters in films assembled from redispersed S‐MXene. After 180 days of storage, the films retained a conductivity of 6700 S cm^−1^ (Figure 4b), in contrast to traditional semi‐solid MXene, whose conductivity declined to 4200 S cm^−1^. Infrared emissivity also remained exceptionally low at 0.12 (Figure 4c), nearly unchanged from the fresh state, whereas conventional semi‐solid MXene increased to 0.26. These results demonstrate that S‐MXene preserves excellent infrared camouflage even after prolonged storage. As shown in Figure 4d, the S‐MXene film maintained low infrared emission when covering a human palm or a centrifuge tube containing hot water, with the recorded surface temperature nearly indistinguishable from the ambient background. The film also exhibited stable low‐emissivity behavior across different heating platforms (Figure S17, Supporting Information) and maintained robust camouflage under both moderate (Figure 4e) and elevated temperatures (Figure 4f). Furthermore, diverse patterns printed using high‐concentration S‐MXene slurries displayed outstanding infrared stealth performance (Figure S18, Supporting Information). Taken together, these results highlight that S‐MXene significantly outperforms conventional semi‐solid MXene in long‐term redispersibility and oxidative resistance. Since hydrolysis is widely recognized as the primary trigger of MXene oxidation, increasing solid content to reduce interlayer water remains an effective stabilization strategy. Importantly, while both S‐MXene and conventional semi‐solid MXene retain small amounts of interlayer water, hence not entirely eliminating oxidation risk, S‐MXene demonstrates markedly superior stability. This suggests the existence of a unique protective mechanism inherent to the LNC‐mediated structure.

To elucidate how LNC endows semi‐solid MXene with long‐term reversible redispersibility and oxidative stability, we systematically examined its role at the nanosheet interface. DFT calculations reveal that LNC possesses a stronger affinity for water molecules than MXene itself (Figure 4g), enabling it to preferentially capture interlayer water. This reduces direct water‐MXene contact and forms a stable barrier that suppresses hydrolysis‐driven oxidation. In addition, this barrier impedes oxygen diffusion into the interlayers, thereby further enhancing stability. Together, these effects constitute a dynamic protection mechanism for semi‐solid MXene. Molecular dynamics simulations further probed the amphiphilic interactions of LNC with MXene. Analysis of hydrogen‐hydrogen distances showed that hydrophilic groups of LNC remain significantly closer to the MXene surface than hydrophobic groups (Figure 1c; Videos S1 and S2, Supporting Information), confirming attractive adsorption of the former and repulsive interactions of the latter. This dual effect allows LNC to firmly anchor onto MXene nanosheets while dynamically balancing interlayer spacing. Based on these insights, we propose a mechanistic model for LNC‐mediated interlayer modulation (Figure 4h). During spray drying, LNC segments coat MXene surfaces to form a confined isolation layer, buffering the capillary compression forces generated by rapid solvent evaporation and preventing irreversible nanosheet restacking. Simultaneously, LNC's strong water‐retention capability preserves continuous interlayer channels, enabling rapid and complete redispersion upon rehydration. In contrast, spray‐dried pure MXene undergoes excessive dehydration, which drives strong hydrogen bonding and van der Waals interactions, and may even trigger graphene oxide‐like crosslinking,^[^ ^21^ ^]^ resulting in permanent loss of redispersibility. We further compared LNC with cellulose nanofibers (CNF), which contain more abundant ─OH groups^[^ ^22^, ^23^, ^24^ ^]^ and stronger intrinsic water‐locking capacity.^[^ ^25^ ^]^ Unlike LNC, MXene dispersions containing 1 wt.% CNF failed to preserve redispersion after spray drying (Figure S19, Supporting Information). This behavior is attributed to the uniform distribution of ─OH functional groups along CNF chains, which promotes excessive nanosheets compression during drying and yields interlayer forces too strong to overcome. By contrast, the amphiphilic nature of LNC confers a unique balance of adsorption and repulsion, enabling redispersion at extremely low loadings (0.5–2 wt.%). Therefore, LNC, low‐cost and biomass‐derived, emerges as one of the few materials capable of driving rapid transformation of dilute MXene dispersions into semi‐solid form while maintaining both long‐term redispersibility and enhanced mechanical integrity, without compromising MXene's intrinsic physicochemical properties.

It is important to emphasize that the LNC‐mediated interlayer chemical modulation of MXene, and the consequent redispersibility of spray‐dried powders, exhibits a distinct time‐dependent effect, reminiscent of the water‐assisted welding phenomenon previously reported in MXene films.^[^ ^26^ ^]^ As shown in Figure 4i, MXene dispersions containing different amounts of LNC (0.5, 1, and 2 wt.%) could all be successfully spray‐dried into powders and redispersed into a semi‐solid structure within ≈2 h. However, beyond this time‐dependent period, their redispersibility progressively diminished (Figure S20, Supporting Information). This observation highlights the necessity of promptly converting spray‐dried powders into a semi‐solid state, in which excellent redispersibility can be preserved for up to 180 days. The underlying origin of this time‐dependent effect is most likely associated with the fluffy morphology of fully solid MXene powders obtained through spray drying (Figure 4j; Figure S21, Supporting Information). LNC‐mediated modulation significantly affects the interlayer spacing, but over time, the confined water molecules gradually escape into the atmosphere. Interestingly, once the powders are converted into semi‐solid MXene within this time‐dependent period, the water molecules entrapped by LNC between the nanosheets are much less likely to evaporate. Instead, they engage in synergistic interactions with the 2D MXene framework, resisting moisture loss. As a result, LNC promotes the formation of a stable and ordered planar hydrogen‐bond network across the interlayers, effectively inducing a more compact and regular stacking of MXene nanosheets. As shown in Figure 4k, the interlayer arrangement of MXene in the S‐MXene film is highly ordered and dense (f = 0.812, where f is the Hermans orientation factor), in sharp contrast to the wrinkled and porous structure of pure MXene films (f = 0.648). This enhanced structural order directly underpins the superior mechanical performance of S‐MXene film compared to pure MXene film (Figure S12b). At the same time, the water‐barrier effect provided by LNC prevents direct contact between interlayer water and the MXene nanosheets, thereby effectively suppressing hydrolysis‐induced oxidation. This interpretation is further supported by the negligible changes in both conductivity (Figure 4b) and infrared emissivity (Figure 4c) of S‐MXene film after 180 days of storage. Notably, the dense interlayer architecture also serves as a robust shield against external moisture infiltration, thereby extending the practical service life of MXene film under operational conditions.

**Figure 5:**
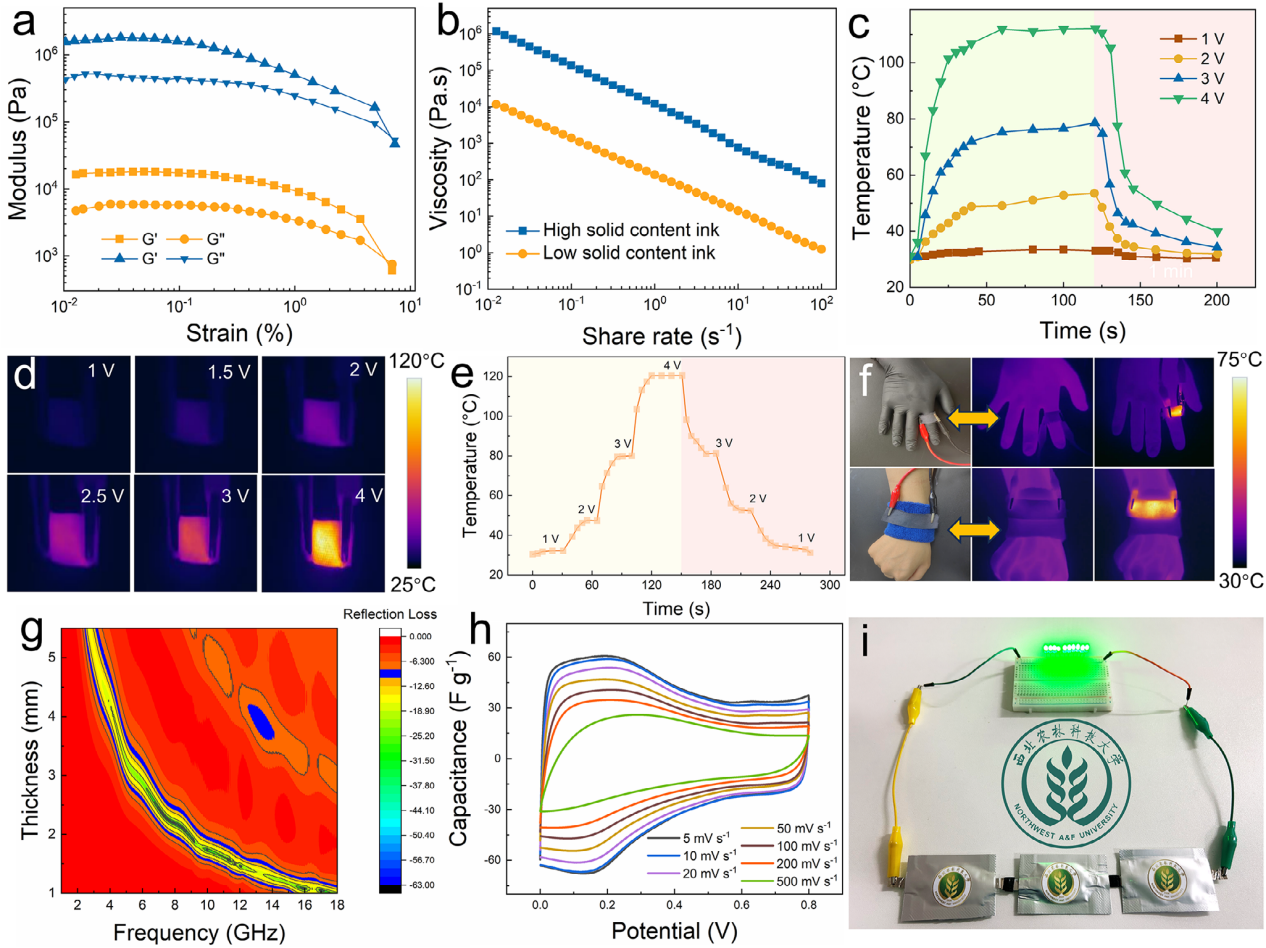
a) Relationship between storage modulus (G') and loss modulus (G*””*) of the ink after the transformation of S‐MXene at different shear stresses, and b) Viscosity changes at different shear rates. c) Time‐temperature curves and d) Infrared thermal images of conductive fabric at different input voltages. e) Linear relationship between current and voltage of the conductive fabric. f) Demonstration of wearable heating using conductive fabric at 2 V. g) Planar view of the EM RL for structurally fixed MXene powder. h) CV curves of the symmetrical supercapacitor assembled with structurally fixed MXene powder, and i) Demonstration of three series‐connected supercapacitors powering an LED light for extended periods.

Given the pronounced time‐dependent effect associated with LNC‐mediated interlayer modulation of MXene, this phenomenon offers unique opportunities for the versatile utilization of spray‐dried MXene powders across diverse application scenarios. Within the time‐dependent period, MXene powder can be rapidly rehydrated into semi‐solid MXene, preserving exceptional long‐term redispersibility and stability, thereby serving as an ideal precursor for a broad range of MXene‐based assemblies. As shown in **Figure** **5**a, the ink derived from semi‐solid MXene exhibits rheological behavior comparable to that of pristine dispersions, characterized by high storage and loss modulus and a pronounced decrease in apparent viscosity with increasing shear rate (Figure 5b), indicative of shear‐thinning behavior well suited for printing and coating applications.^[^ ^27^, ^28^, ^29^ ^]^ The ink was employed to coat cotton fabrics, whose electrical conductivity was validated through the linear relationship between saturation temperature and the square of the driving voltage (Figure S22a, Supporting Information), as well as the current–voltage dependence (Figure S22b, Supporting Information), both confirming excellent Ohmic conduction. Figure 5c,d presents the time‐temperature curves and corresponding infrared images of the fabric under different applied voltages. Increasing the voltage from 1 to 4 V led to a rapid rise in surface temperature from 32.3 to 115 °C within seconds, followed by an equally rapid return to ambient temperature upon power removal. Moreover, real‐time temperature tuning and rapid stabilization could be readily achieved by voltage adjustment (Figure 5e), demonstrating excellent thermal management capability. Durability under prolonged operation is equally critical for heating devices. As shown in Figure S23a (Supporting Information), when powered continuously at 2 V for 30 min, the fabric maintained a stable temperature of ≈52 °C, evidencing reliable thermal stability. Cyclic heating‐cooling tests further confirmed uniform and reproducible temperature responses (Figure S23b, Supporting Information). Integration into wearable platforms such as gloves and wristbands (Figure 5f) markedly enhanced heat generation and diffusion, fulfilling practical demands for personal thermal management. Beyond the time‐dependent period, however, the wrinkled, floral‐like MXene powder morphology becomes irreversibly fixed, precluding redispersion and effectively locking its microstructure. Such structure‐fixed MXene powders retain exceptional utility: when incorporated as fillers or assembled into defined architectures, their internal structure remains stable, ensuring robust performance. For instance, these powders exhibit outstanding microwave absorption properties, with a minimum reflection loss of ‐56 dB (Figure 5g). Furthermore, their enlarged accessible surface area and fully exposed active sites endow the supercapacitor (Figure S24, Supporting Information) assembled from this powder with significantly enhanced rate capability (Figure 5h) relative to the pure MXene film (Figure S25, Supporting Information), while maintaining nearly unchanged capacitance even after 10 000 cycles (Figure S26, Supporting Information). Notably, three supercapacitors connected in series were capable of stably powering an LED for extended durations (Figure 5i; Video S3, Supporting Information). Collectively, these findings demonstrate that the time‐dependent interlayer modulation imparted by LNC not only enables reversible processing of MXene but also provides a versatile platform for stabilizing structure‐fixed powders, thereby unlocking the full performance potential of MXene across a wide spectrum of functional applications.

MXene materials have demonstrated immense application potential across various fields, but their truly game‐changing core uses remain to be further identified.^[^ ^30^ ^]^ Although this work does not target specific downstream applications, the developed semi‐solid MXene technology is poised to play a transformative role in bridging critical segments of the MXene supply chain, including its initial dispersion, storage, transportation, and subsequent processing. Given that MXene possesses nearly 100% photothermal conversion efficiency,^[^ ^31^ ^]^ photothermal applications could provide a practical breakthrough pathway from laboratory research to commercial deployment. To underscore the importance of semi‐solid MXene in establishing an integrated industrial chain and accelerating commercialization, we further explored its potential in the photothermal domain. Solar‐driven interfacial water evaporation is an emerging green water‐treatment strategy,^[^ ^32^ ^]^ in which solar energy alone is harvested by photothermal materials to convert sunlight to heat; this heat is then localized at the air‐water interface to drive efficient evaporation, with the generated vapor condensed to yield clean freshwater. As a renewable, distributed approach, it offers a sustainable solution to freshwater scarcity, especially in remote and off‐grid regions.^[^ ^33^, ^34^, ^35^ ^]^ The central challenge lies in developing photothermal materials with high conversion efficiency and operational stability. Compared with traditional metal‐based,^[^ ^36^ ^]^ carbon‐based,^[^ ^37^ ^]^ and conjugated polymer‐based^[^ ^38^ ^]^ photothermal materials, MXene combines superior biocompatibility^[^ ^3^, ^39^ ^]^ with a broader solar absorption bandwidth,^[^ ^40^ ^]^ enabling more efficient utilization of the full solar spectrum. Its inherently low infrared emissivity reduces radiative heat loss, further enhancing energy efficiency, while highly tunable surface chemistry and interlayer structure facilitate functional modification. These attributes position MXene as a compelling candidate for solar‐driven interfacial water evaporation, providing a new materials platform for efficient and stable distributed water‐treatment systems.

**Figure 6:**
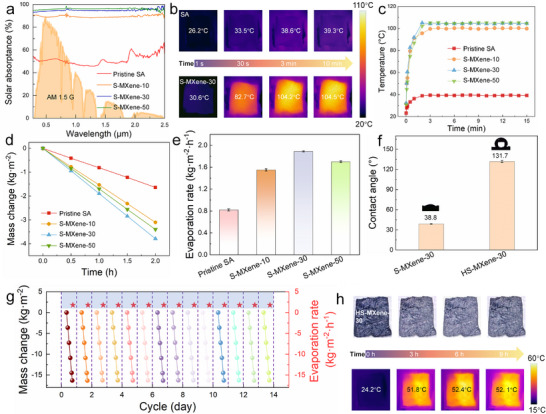
a) UV–vis–NIR absorption spectra of pure SA and S‐MXene/SA composite hydrogels. b) Infrared thermal images of dry SA and S‐MXene‐30 gels under one sun irradiation. c) Temperature rise curves of pure SA and S‐MXene/SA composite hydrogels under one sun and dry conditions. d) Mass changes of pure water, and e) evaporation rate of pure SA and S‐MXene/SA composite hydrogels (*n* = 5; error bars indicate the standard deviation). f) Water contact angle of S‐MXene‐30 gel before and after hydrophobic treatment (*n* = 5; error bars indicate the standard deviation). g) Water mass change of HS‐MXene‐30 composite gel in 3.5 wt.% NaCl solution under 14 days of cyclic exposure to 9 h of daily sunlight. h) Optical image and infrared thermal image of the HS‐MXene‐30 gel surface during the 14th cycle.

Although neither MXene nor S‐MXene alone achieves efficient full‐spectrum absorption across the ultraviolet‐visible‐near‐infrared (UV–Vis–NIR) range (Figure S27, Supporting Information), incorporating them as high‐performance photothermal enhancers into host matrices offers substantial potential to markedly improve solar absorption and photothermal conversion of composites. To assess the applicability of S‐MXene for solar‐driven interfacial water evaporation, we embedded S‐MXene into a sodium alginate (SA) gel and prepared S‐MXene/SA composite gels with excellent photothermal performance. As shown in **Figure** **6**a, within the UV–Vis‐NIR range, the solar absorption of pure SA gel is only 50.12%, whereas increasing the S‐MXene content significantly raises the density of photothermal active sites in the composite. The absorption rates for S‐MXene‐30 (30 wt.% MXene) and S‐MXene‐50 (50 wt.% MXene) composite gels reached 94.52% and 95.90%, respectively. This outstanding full‐spectrum solar light absorption ability provides a key condition for achieving efficient photothermal conversion. Under a standard solar irradiance intensity (1 Sun), we evaluated the photothermal conversion of the dry composite gels. As shown in Figure 6b,c and Figure S28 (Supporting Information), the S‐MXene composite gels exhibited substantially faster heating rates and rapidly reached thermal equilibrium compared with pure SA gel, highlighting their superior photothermal conversion efficiency. This ensures more effective utilization of solar energy and provides sufficient thermal input for subsequent interfacial water evaporation. We further assessed the pure‐water evaporation performance of the gels under 1 Sun irradiance (Figure S29, Supporting Information). Interestingly, the evaporation rate initially increased with S‐MXene content but decreased at higher loadings. The S‐MXene‐30 gel exhibited the best performance, achieving an evaporation rate of 1.89 kg m^−2^ h^−1^, while the rate for S‐MXene‐50 declined slightly to 1.70 kg m^−2^ h^−1^ (Figure 6d,e). This behavior is primarily attributed to severe restacking of S‐MXene nanosheets at higher concentrations, leading to structural densification (Figure S30a, Supporting Information), which impedes water transport and vapor release. By contrast, the internal structure of S‐MXene‐30 was relatively loose and porous (Figure S30b, Supporting Information), which facilitated water transport and vapor release, resulting in better water evaporation performance. Notably, the photothermal conversion and evaporation performance of S‐MXene‐10 surpass those of the SA composite with a higher carbon black loading (CB‐16, 16 wt.% carbon black, particle size 10–15 nm) (Figure S31, Supporting Information), highlighting the distinctive advantage of MXene in solar‐driven interfacial evaporation.

Although both MXene and LNC exhibit strong hydrophilicity, enabling rapid water uptake and efficient transport within composite gels, thereby ensuring a stable and timely water supply for solar‐driven interfacial evaporation, the hydrophilic interface (Figure 6f) is prone to salt accumulation during long‐term seawater desalination. This fouling severely compromises material stability and practical application. To address this limitation and enhance performance under realistic conditions, we hydrophobically modified the surface of the S‐MXene‐30 composite gel with perfluorosilane, yielding a Janus‐structured functional material.^[^ ^41^, ^42^ ^]^ As shown in Figure 6f, the water contact angle of the hydrophobically modified S‐MXene‐30 (HS‐MXene‐30) composite gel surface significantly increased to 131°. We subsequently conducted continuous long‐term cyclic evaporation tests using HS‐MXene‐30 composite gel in 3.5 wt.% NaCl artificial seawater for 14 days, under simulated natural day‐night cycles (9 h illumination, 15 h darkness). As shown in Figure 6g, the HS‐MXene‐30 composite gel maintained a stable evaporation rate of ≈1.81 kg m^−2^ h^−1^ throughout the test period, with no discernible decline. Furthermore, optical and infrared thermal images taken after 9 h of continuous illumination on day 14 (Figure 6h) revealed no visible salt crystallization, confirming excellent salt‐rejection capability alongside robust photothermal conversion stability. Together, these results demonstrate that HS‐MXene‐30 composite gel possesses excellent interfacial evaporation stability and durability, fully meeting the practical requirements for seawater desalination. This provides compelling experimental evidence supporting the practical deployment of S‐MXene in solar‐driven interfacial water evaporation technologies.

## 3 Conclusion

In summary, this work proposes a lignin‐containing nanocellulose (LNC)‐mediated strategy for interlayer chemical modulation of MXene, enabling precise control over interlayer architecture and interface chemistry through competitive interactions among MXene nanosheets. This approach facilitates the rapid and scalable production of semi‐solid MXene. The semi‐solid MXene demonstrates excellent long‐term reversible redispersibility and oxidative stability, remaining fully redispersible into monolayer MXene even after 180 days of storage, while preserving exceptionally high electrical conductivity. Moreover, by flexibly adjusting the time‐dependent period after spray‐drying, a controllable transition between dynamically reversible and permanently fixed MXene powder can be achieved, thereby expanding their application landscape across conductive textiles, electromagnetic wave absorption, supercapacitors, and solar‐driven interfacial water evaporation. This work effectively addresses long‐standing barriers to the industrial‐scale deployment of MXene, including oxidation vulnerability, high transportation costs, and reprocessing challenges, and establishes a crucial technological bridge from laboratory‐scale synthesis to real‐world commercialization.

## 4 Experimental Section

### Preparation of Monolayer MXene (Ti_3_C_2_T_x_)

Monolayer MXene (Ti_3_C_2_T*_x_*) dispersion was synthesized using an improved minimum intensity interlayer delamination protocol for etching Ti_3_AlC_2_ MAX phase, as previously established by the group.^[^ ^43^ ^]^ In a typical procedure, 80 g of LiF (Aladdin) was dissolved in 1375 mL of 12 m HCl (Xilong Chemical) within a custom‐designed polypropylene reactor. Subsequently, 50 g of Ti_3_AlC_2_ MAX powder (Harbin Zhisu Future Technology Co., Ltd.) was added, and the mixture was stirred at 55 °C for 24 h. After completion, the reaction product was repeatedly washed with deionized water until the pH stabilized at ≈6, yielding multilayer MXene. The obtained multilayer MXene was then subjected to our previously reported high‐temperature ultrasonic exfoliation method,^[^ ^9^ ^]^ and the supernatant was collected by centrifugation to obtain the final monolayer MXene dispersion.

### Synthesis of Lignin‐Containing Nanocellulose (LNC)

Lignin‐containing nanocellulose (LNC) was prepared via an improved ternary deep eutectic solvent (TDES) pretreatment combined with microfluidic mechanical defibrillation, as previously developed by our group.^[^ ^17^ ^]^ Briefly, TDES was first obtained by mixing choline chloride (ChCl), lactic acid (La), and p‐toluenesulfonic acid (p‐TsOH) at a molar ratio of 2:10:1, followed by heating in an oil bath at 80 °C under constant stirring for 2 h until a transparent solution was formed. The resulting TDES was reheated to 80 °C, and poplar wood powder was introduced at a solid‐to‐liquid ratio of 1:50. The reaction was maintained under magnetic stirring for 3 h. Upon completion, the mixture was cooled to room temperature, allowed to stand for 10 min, and quenched with deionized water. The solid residues were collected by centrifugation, washed thoroughly by filtration to remove residual solvent and impurities, and subsequently diluted with deionized water to form a lignocellulose suspension. This suspension was then processed three times using a microfluidic homogenizer at 20 000 psi to further exfoliate and fibrillate, ultimately yielding the final LNC dispersion.

### Preparation of Semi‐Solid MXene (S‐MXene)

Monolayer MXene dispersion (8 mg mL^−1)^ was thoroughly mixed with LNC dispersion to achieve a final LNC mass fraction of 1 wt.%. The resulting mixture was subjected to spray drying under controlled conditions, with an inlet temperature of 180 °C, outlet temperature of 80 °C, operating power of 5.5 kW, and a nozzle diameter of 1 mm. The resulting powder was then rehydrated with a small amount of water and kneaded to obtain semi‐solid MXene, hereafter denoted as S‐MXene. The same procedure was used to prepare S‐MXene with LNC mass fractions of 0.5 and 2 wt.%. Unless otherwise specified, S‐MXene refers to the sample prepared with 1 wt.% LNC.

### Preparation of S‐MXene/Sodium Alginate (SA) Composite Gel

Sodium alginate (SA) was first dissolved in deionized water to prepare an SA solution, which was then uniformly mixed with dispersed S‐MXene to form a homogeneous mixture. The mixture was freeze‐dried to yield the composite gel, denoted as S‐MXene‐x, where x represents the mass percentage of MXene. The gel was cut into small pieces and immersed in 3% CaCl_2_ solution for 12 h to induce ionic crosslinking and solidification. The resulting gel was subsequently washed several times with deionized water and air‐dried at room temperature. For surface modification, 1H,1H,2H,2H‐perfluorodecyltrimethoxysilane was dissolved in anhydrous ethanol and uniformly sprayed onto the upper surface of the composite gel with a spray gun. After air‐drying at room temperature, the spraying process was repeated two to three times to obtain a hydrophobic Janus‐type gel, denoted as HS‐MXene‐x.

### Characterization Methods

The morphology and microstructure of MXene samples were examined using field emission high‐resolution transmission electron microscopy (TEM, Tecnai G2 F30) and scanning electron microscope (SEM, ZEISS Sigma 300). Atomic force microscopy (AFM) images of the samples in both contact and tapping modes were obtained using Bruker FastScan. X‐ray diffraction (XRD) was performed on a Bruker D8 Advance diffractometer with Cu Kα radiation. X‐ray photoelectron spectroscopy (XPS) was conducted on a Thermo Scientific ESCALAB Xi+ instrument (USA), and Raman spectra were acquired using a Renishaw Invia spectrometer (UK). For Zeta potential measurements, dispersions were diluted to 0.02 mg mL^−1^ and analyzed using a dynamic light scattering (DLS) particle size/potential analyzer. The mechanical properties of films were evaluated with a universal electronic tensile tester. Viscoelastic properties of semi‐solid composites and redispersed suspensions were measured using a rotational rheometer with a 40 mm plate and a 1 mm gap. Water contact angles of the redispersed films were determined using a contact angle analyzer. Electrical conductivity was measured using an ST‐2258C multifunctional digital four‐probe tester. Solar reflectance spectra of MXene samples were recorded with a PerkinElmer Lambda 950 spectrophotometer equipped with a 150 mm integrating sphere. Fourier‐transform infrared (FT‐IR) spectra were collected using a Nicolet iS50 FT‐IR spectrometer (Thermo Fisher) with a gold integrating sphere (model 660–107400) within the 8–13 µm atmospheric window for infrared emissivity measurements. Photothermal performance was assessed using a xenon lamp as the light source, and infrared thermal images were acquired with a Fluke RSE60 thermal imaging camera. Supercapacitor performance of the MXene powder was tested by mixing it with polytetrafluoroethylene (PTFE) emulsion at a 9:1 ratio, coating it onto graphite sheets to form electrodes, and placing two identical electrodes on either side of a polyacrylic acid‐based gel electrolyte. The assembled supercapacitor was then encapsulated. Electromagnetic parameters of MXene powders were determined in the 2–18 GHz frequency range using a vector network analyzer (PNA‐N5244A) via coaxial line measurement.

### DFT Calculation

Density functional theory (DFT) calculations were performed using the Vienna Ab‐initio Simulation Package (VASP),^[^ ^44^ ^]^ with system setup, execution, and post‐analysis facilitated through an integrated graphical interface. The projected augmented wave (PAW) method was employed to describe core‐electrons interactions, while the exchange–correlation energy was treated within the generalized gradient approximation (GGA) using the Perdew‐Burke‐Ernzerhof (PBE) functional.^[^ ^45^ ^]^ A plane‐wave cutoff energy of 500 eV was applied. Both structural optimization and electronic structure calculations were conducted on a 3 × 3 × 1 Monkhorst‐Pack k‐point grid. The conjugate gradient method was used to relax atomic positions until the residual force on each atom was below 0.02 eV/Å, with an energy convergence threshold of 10^−5^ eV. Gaussian smearing with a broadening factor of 0.05 eV was adopted for electronic occupancy.^[^ ^46^ ^]^

### Molecular Dynamics Simulations

Molecular dynamics (MD) simulations were carried out using the GROMACS 2023.1 package.^[^ ^47^ ^]^ MXene and LNC models were constructed via the sobtop method and packed into a cubic simulation box (6 nm per side) using PACKMOL. The systems were solvated with water, and chloride and sodium ions were introduced to ensure charge neutrality. The AMBER14 force field was employed, with RESP charges obtained by optimizing molecules' geometries at the B3LYP level with the 6–31+G(d,p) basis set in Gaussian 09, incorporating the SDM method to account for solvent effects.^[^ ^48^ ^]^ Further refinement of RESP charges and wavefunctions was performed using Multiwfn.^[^ ^49^ ^]^ All simulations were conducted under periodic boundary conditions in three dimensions, with short‐range interactions treated using the Verlet neighbor list (cutoff 1.2 nm). Temperature control was achieved with the modified Berendsen thermostat (V‐rescale in GROMACS), and pressure regulation was implemented via the Berendsen barostat. Long‐range electrostatic interactions were computed using the particle‐mesh Ewald (PME) method. The simulation protocol proceeded stepwise. Energy minimization was first conducted using the steepest descent algorithm, followed by equilibration under the NVT and NPT ensembles (5 ns each). In the production phase, designed to probe the distance variations between MXene and LNC, hydrophilic (─OH) and hydrophobic (─OCH_3_) groups of LNC were aligned along the positive *x*‐axis, with structural optimization performed under positional constraints. MXene was restrained along the *x*‐ and *z*‐axes, allowing motion only along the *y*‐axis. The production run lasted 5 ns, during which parameters such as average density were recorded. Visualization of simulation trajectories, snapshots, and animations was performed using VMD.

## Conflict of Interest

The authors declare no conflict of interest.

## Supporting information



Supporting Information

Supplemental Video 1

Supplemental Video 2

Supplemental Video 3

## Data Availability

The data that support the findings of this study are available from the corresponding author upon reasonable request.;
