# Supplementary material for: Lignin‐Containing Nanocellulose Mediated Interlayer Modulation Unlocks Stable and Redispersible MXene
Source: Adv Sci (Weinh). 2025 Oct 3;12(47):e08665. doi: 10.1002/advs.202508665 (PMC12713015; doi:10.1002/advs.202508665)
Supplement: Supplementary file 1 — Supporting Information [file ADVS-12-e08665-s004.docx]

Supporting Information

Lignin-Containing Nanocellulose Mediated Interlayer Modulation Unlocks Stable and Redispersible MXene

Shuyang He, Zhen Yu, Shan Li, Shijie Lei, Lin Zhu, Ke Zhao, Fangxia Yang,* Ningning Cao,* Yuyan Liu, and Zhimin Fan*

Supporting Figures

**
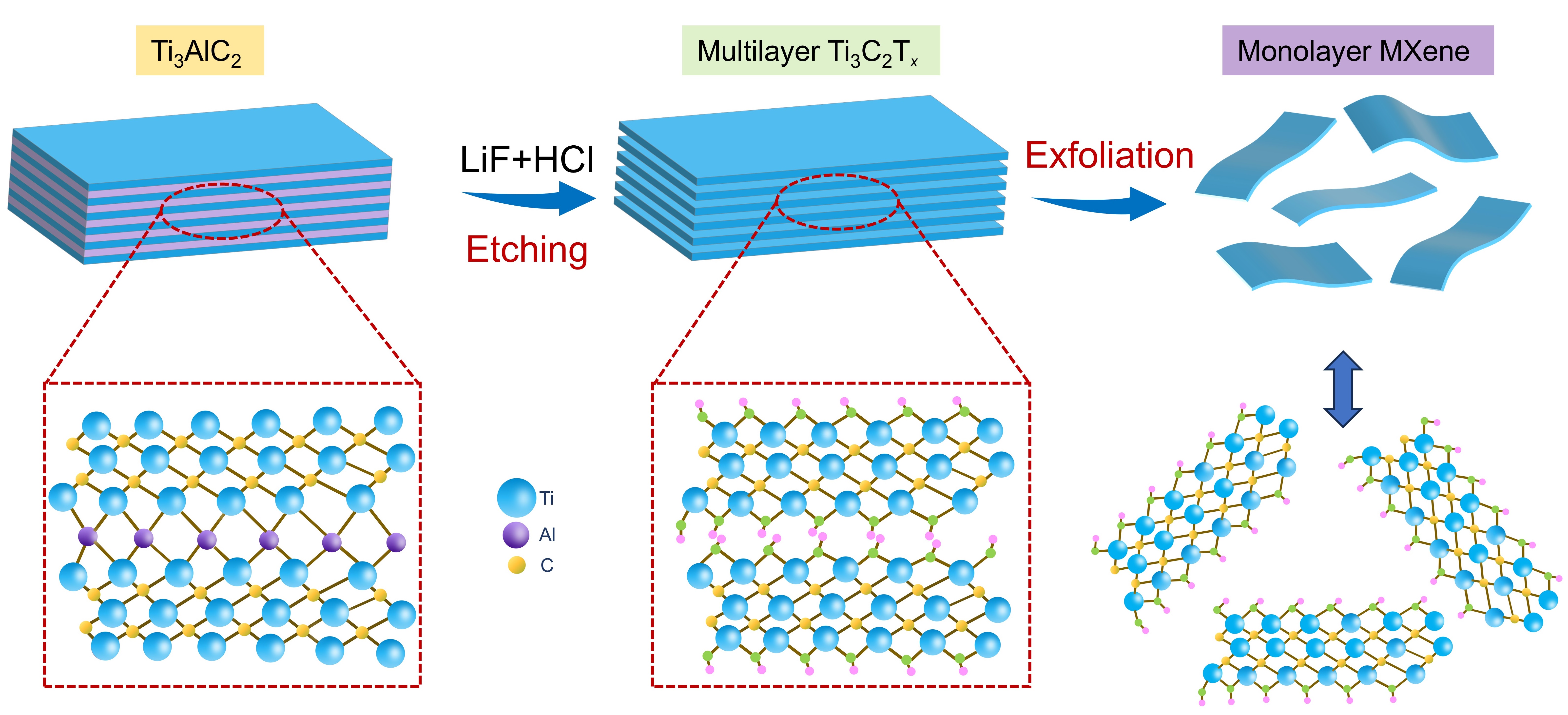
**

**Figure S1**. Illustration of synthesis of the Ti_3_C_2_T*_x_* MXene nanosheets.


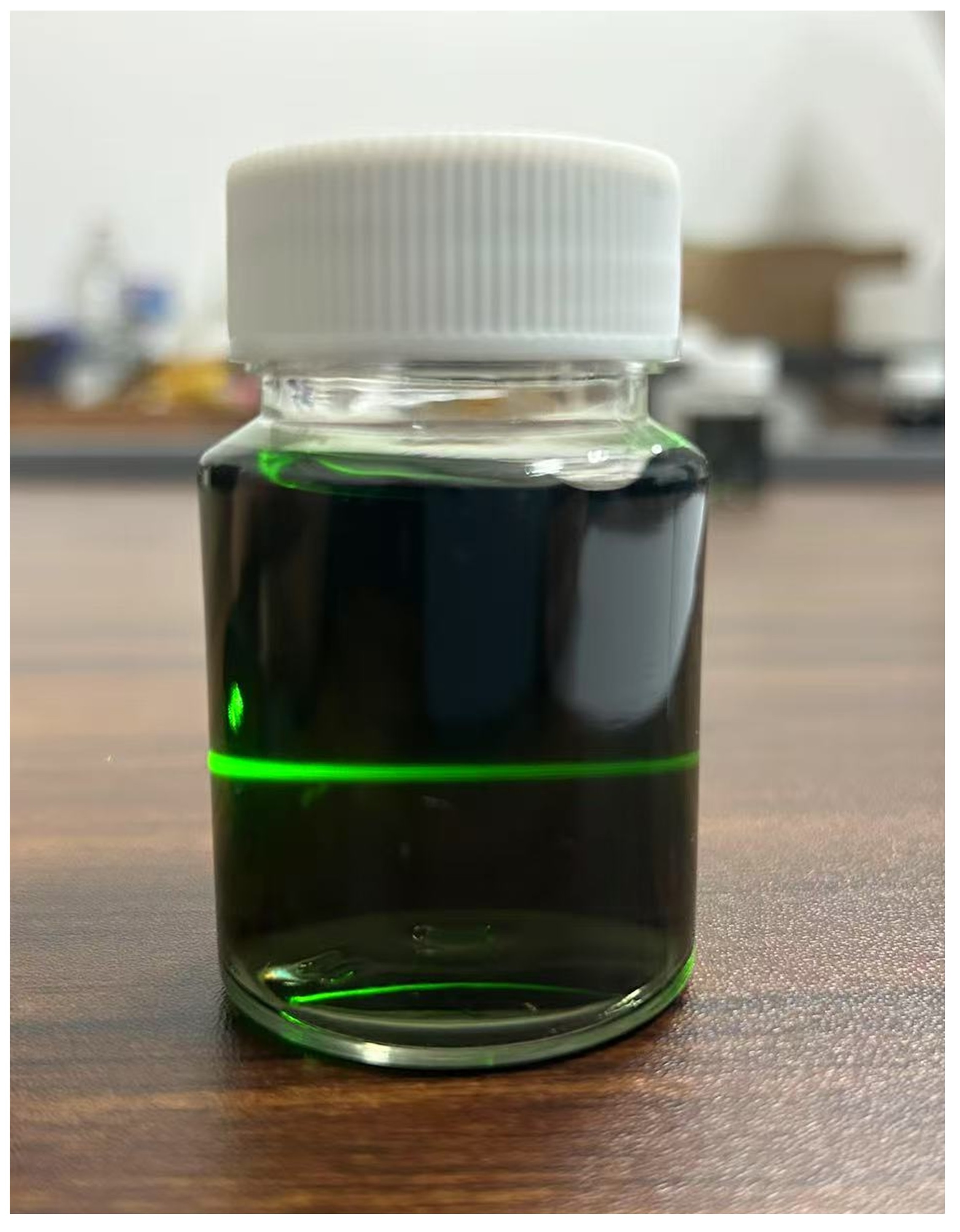


**Figure S2**. Photograph of diluted MXene dispersion.


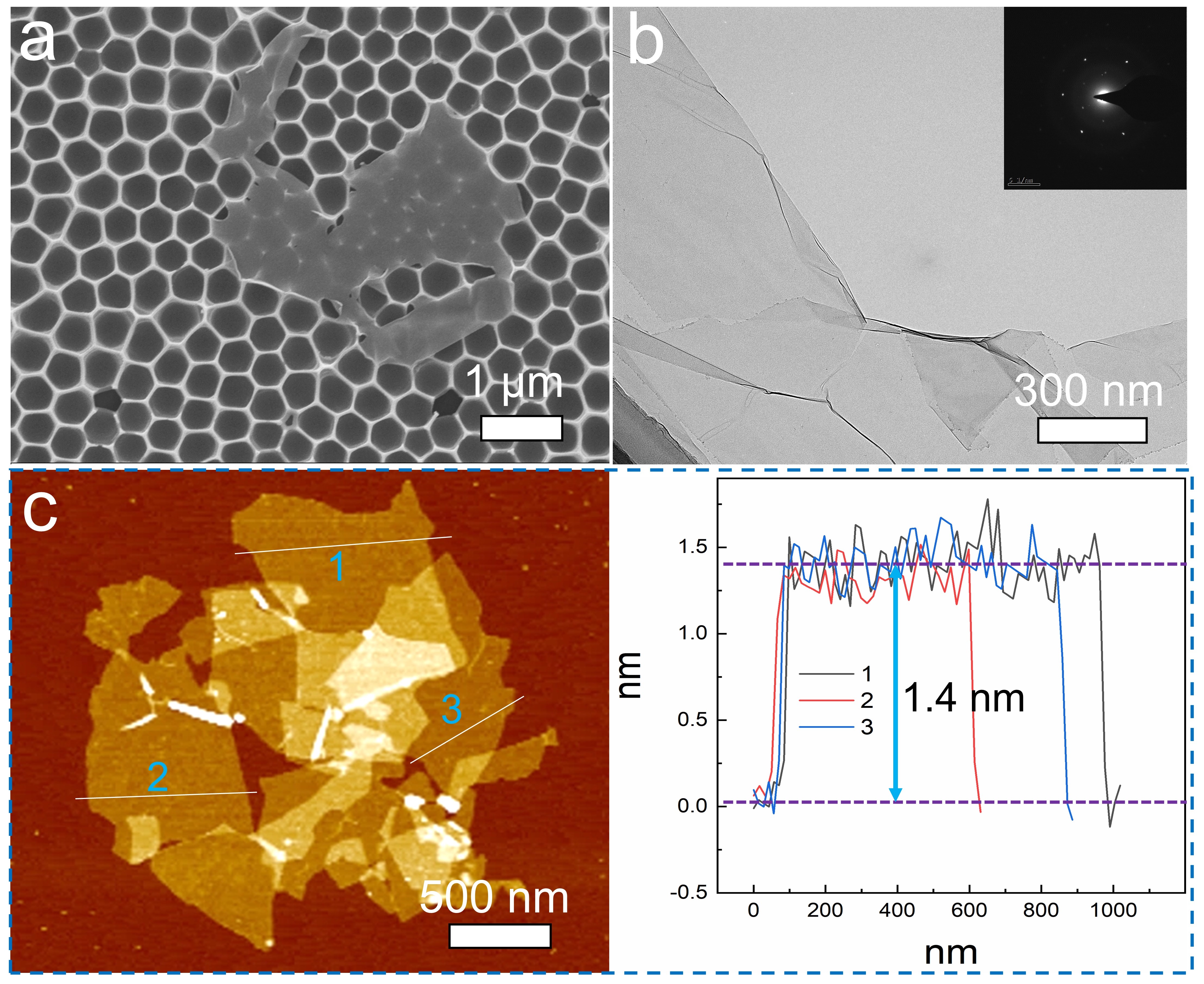


**Figure S3.** (a) SEM image of MXene nanosheets. (b) TEM image and corresponding SAED pattern of MXene nanosheets. (c) AFM images of MXene nanosheets on mica with the corresponding height profiles.


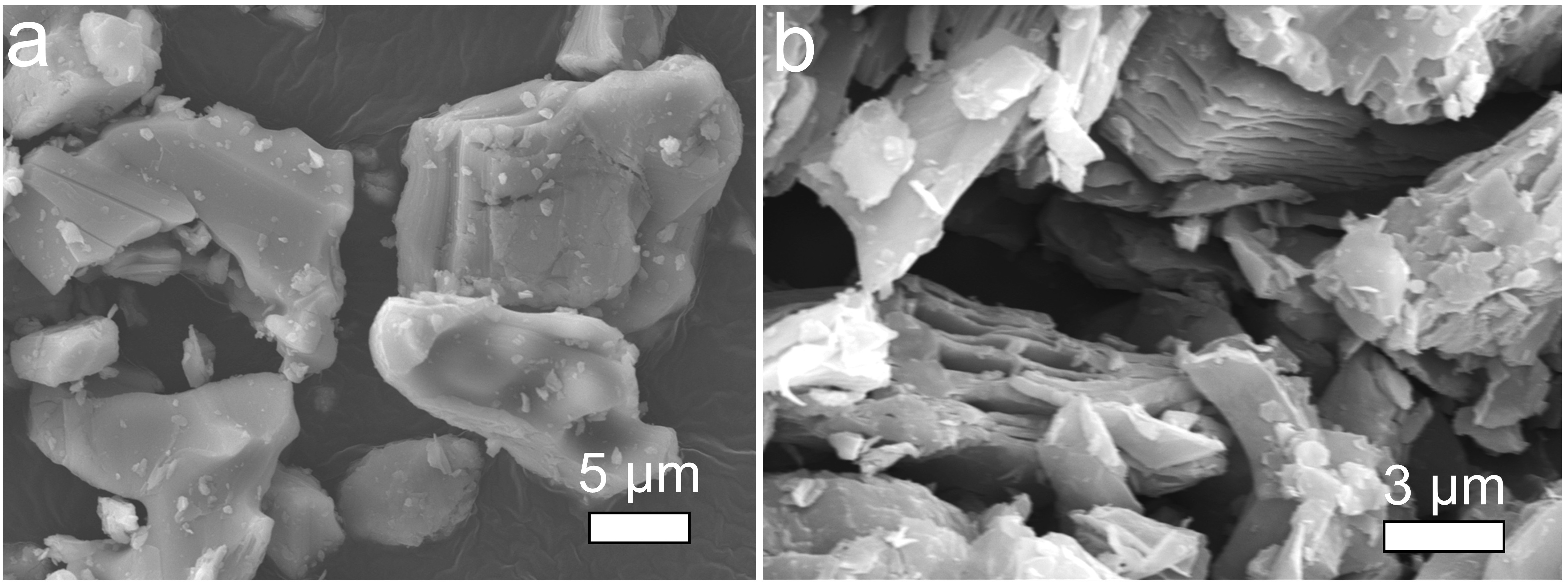


**Figure S4**. SEM images of (a) Ti_3_AlC_2_ MAX and (b) multilayer MXene.


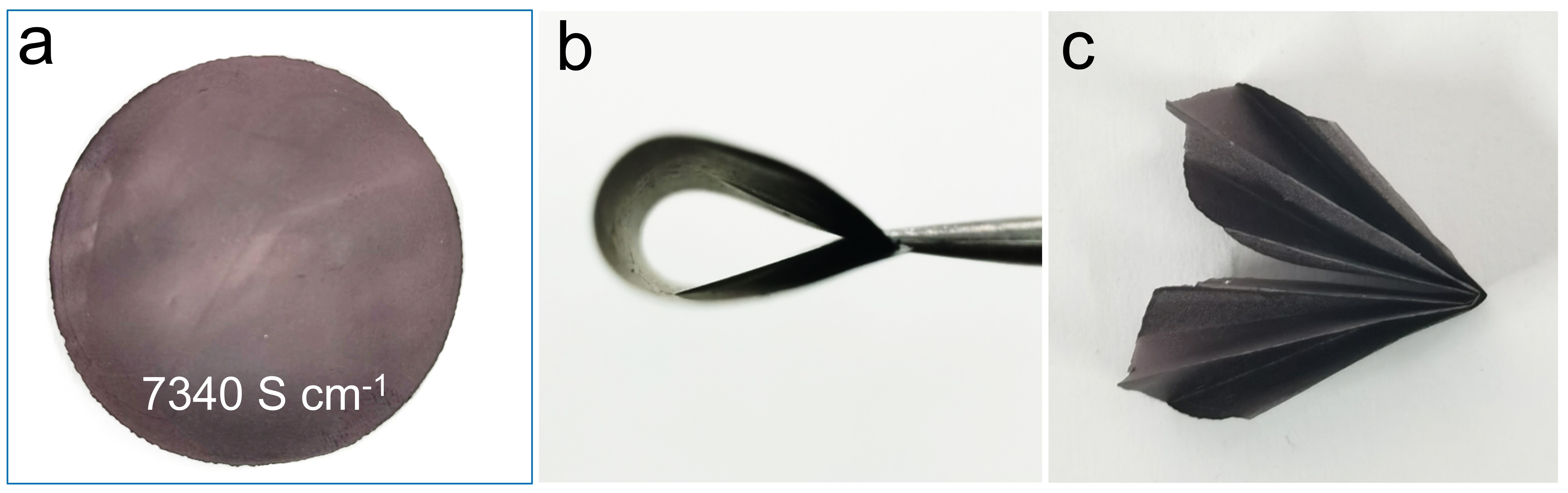


**Figure S5**. (a) Photograph of MXene film. (b) Flexibility of MXene film. (c) Foldability of MXene film.


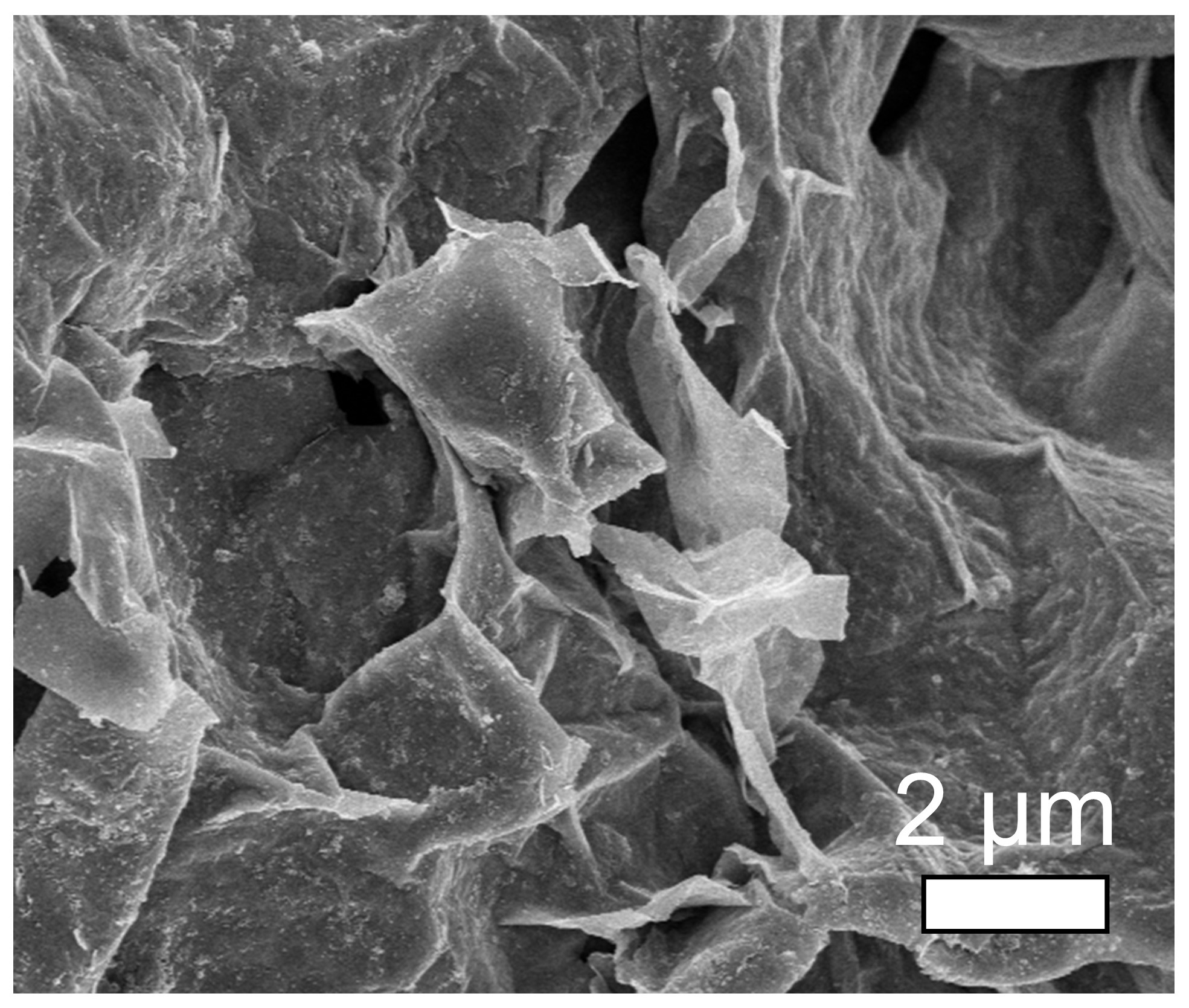


**Figure S6**. SEM image of the internal structure of S-MXene.


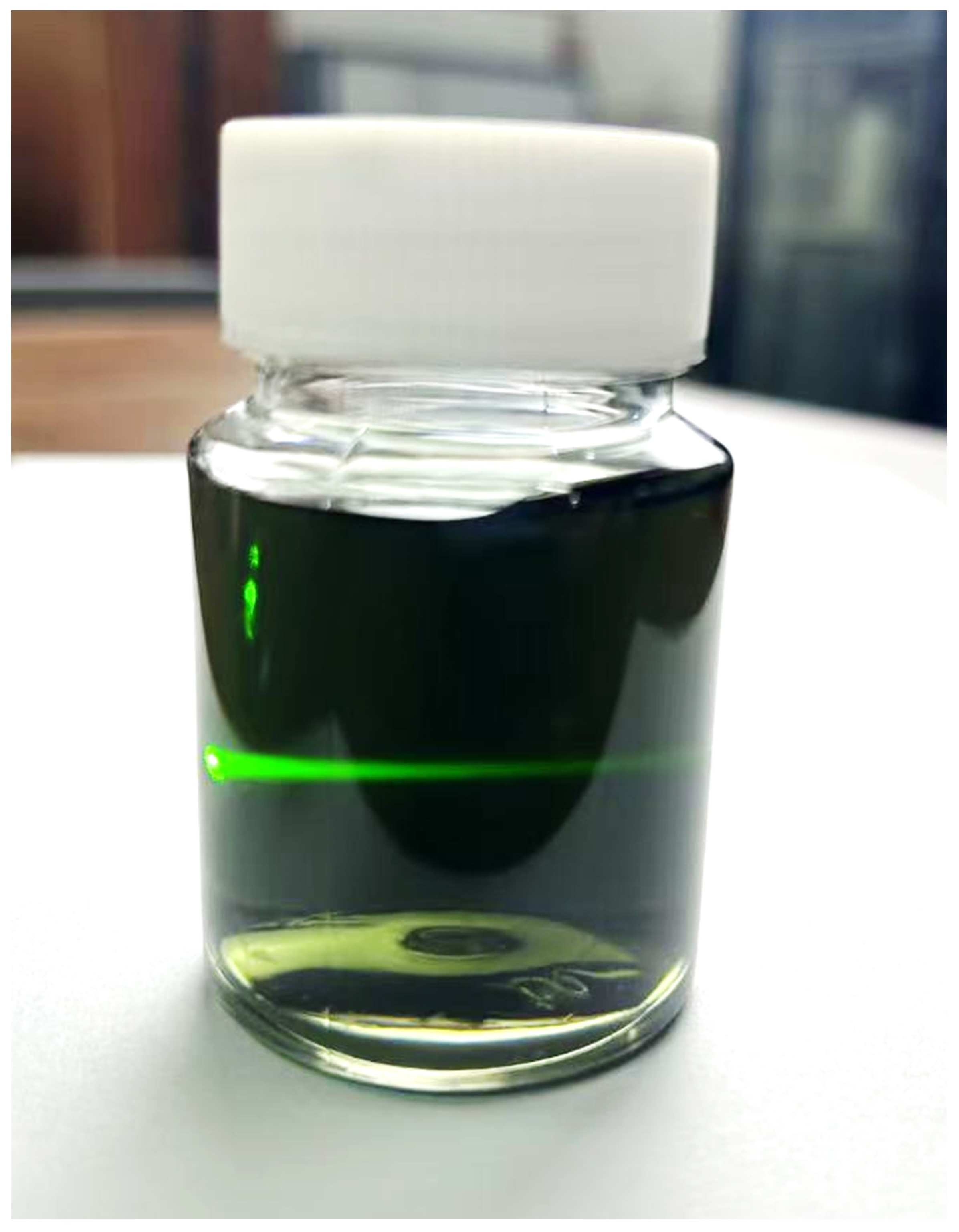


**Figure S7**. Photograph of the diluted dispersion of S-MXene after redispersion.


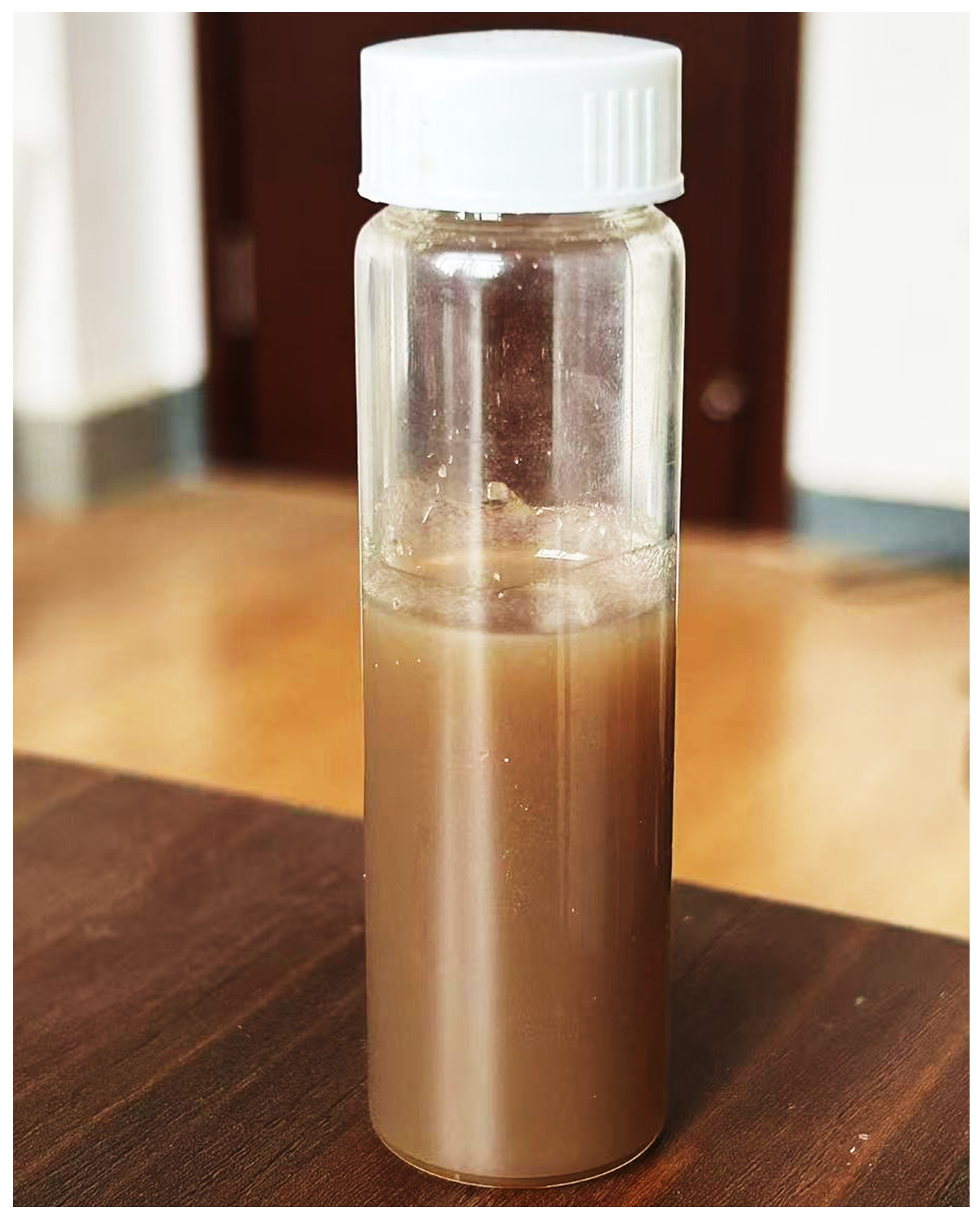


**Figure S8**. Stability demonstration of LNC dispersion after 30 days of storage.


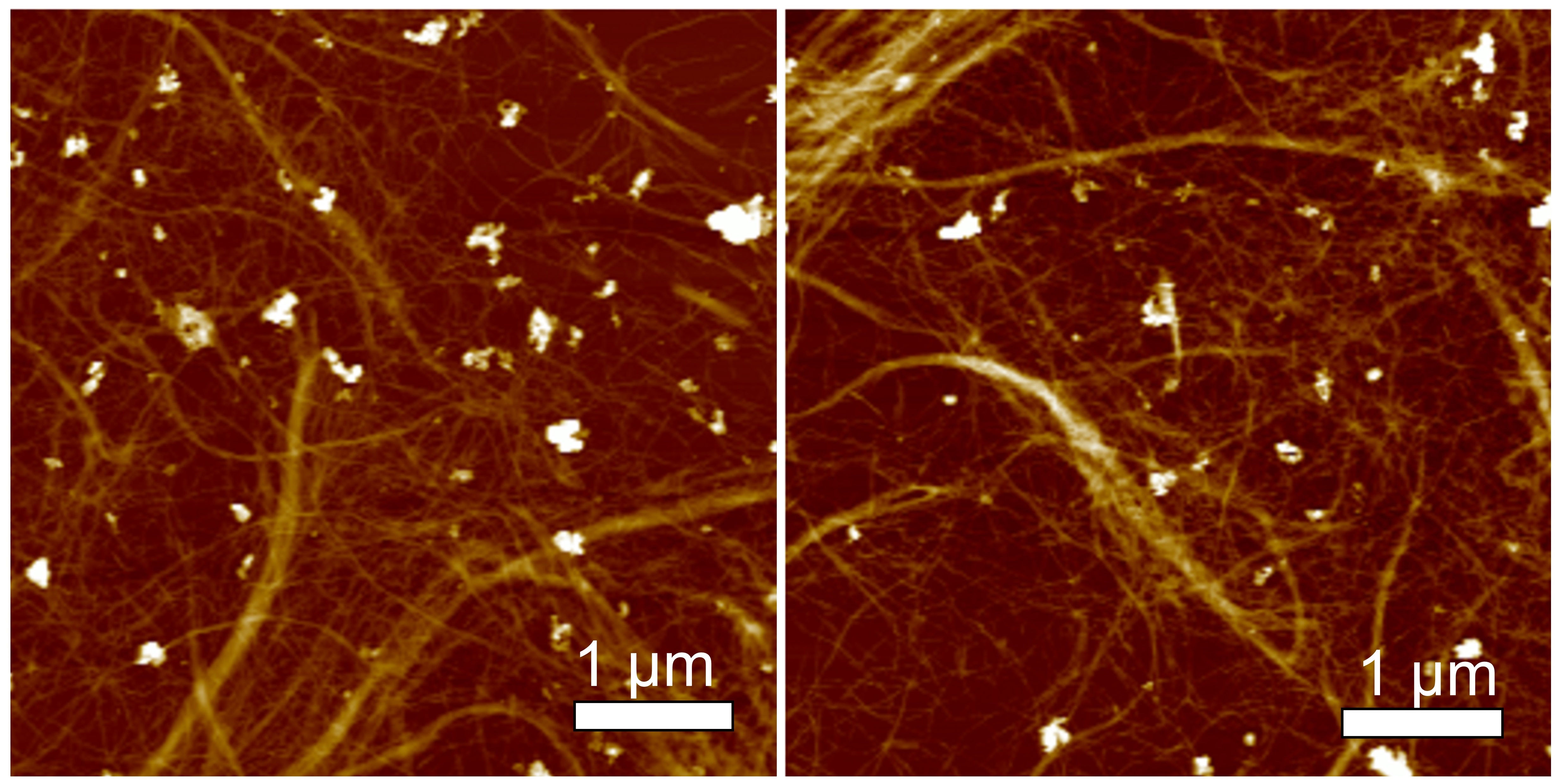


**Figure S9.** AFM images of LNC.


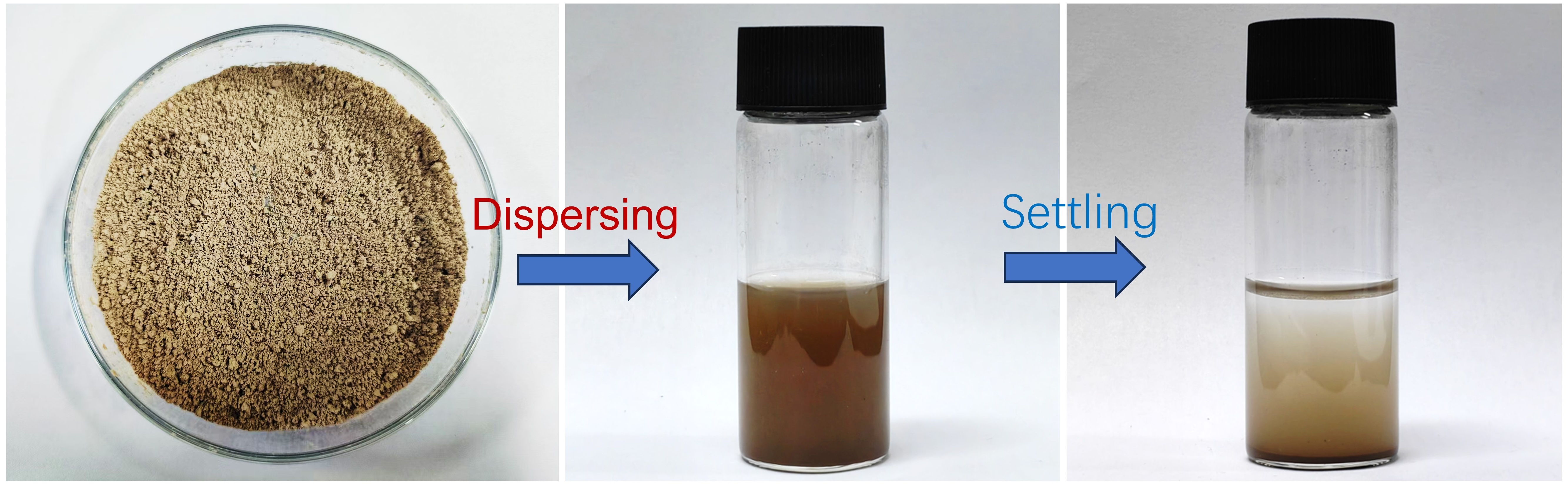


**Figure S10.** Redispersibility demonstration of the powder obtained from spray drying of LNC dispersion.


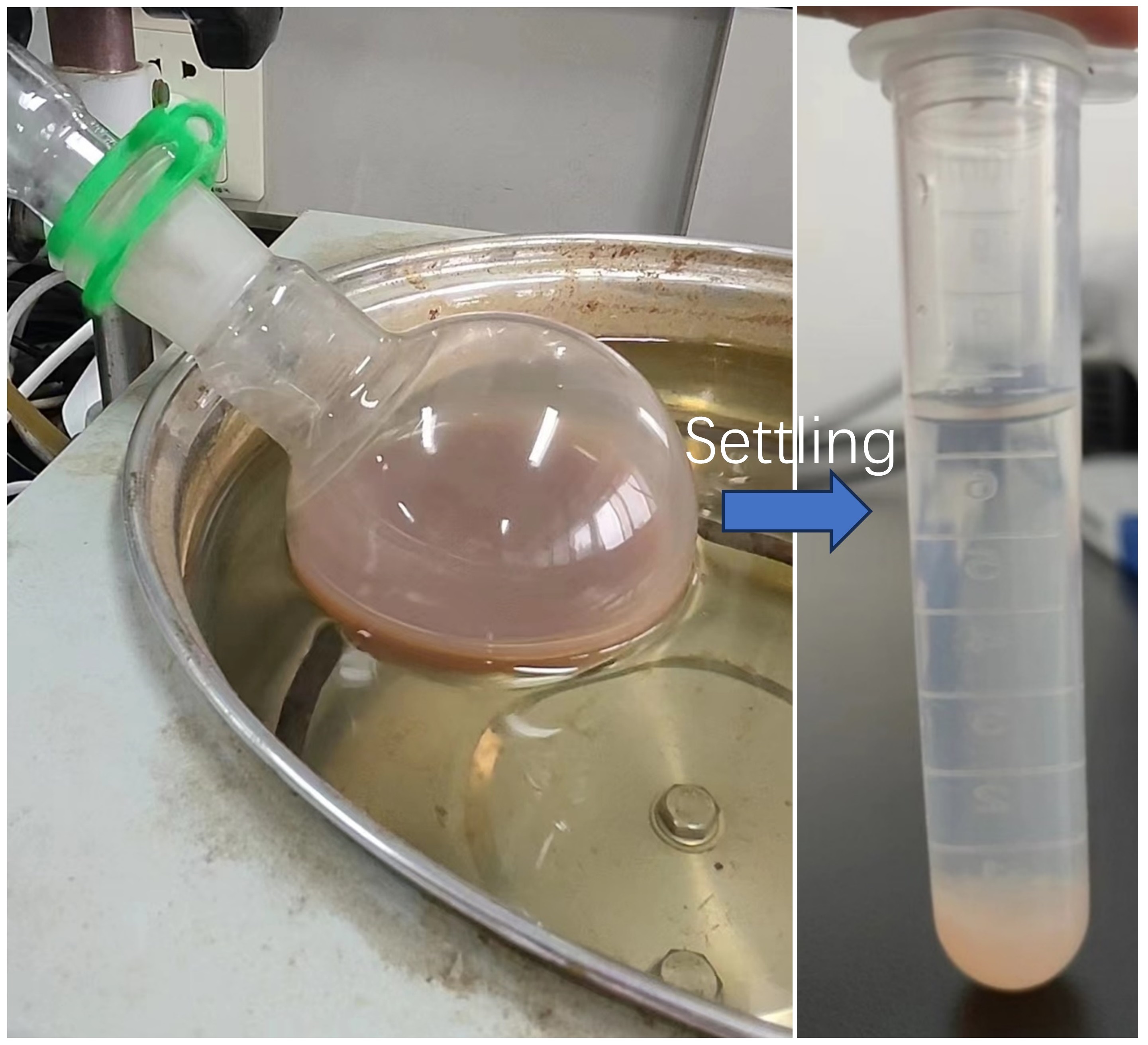


**Figure S11*.*** Demonstration of concentration enhancement and redispersibility of LNC dispersion via distillation.


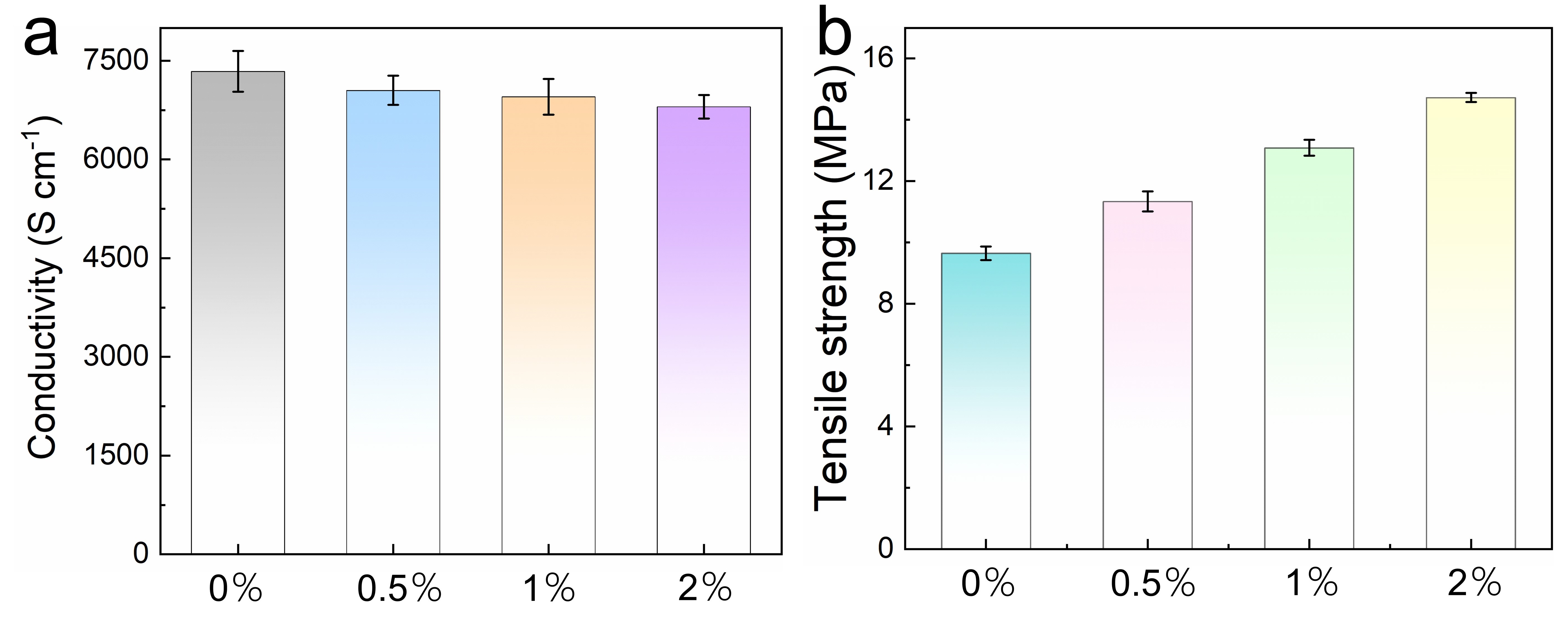


**Figure S12.** Films assembled from redispersed semi-solid MXene: (a) conductivity and (b) tensile strength. (n=5; error bars indicate the standard deviation)


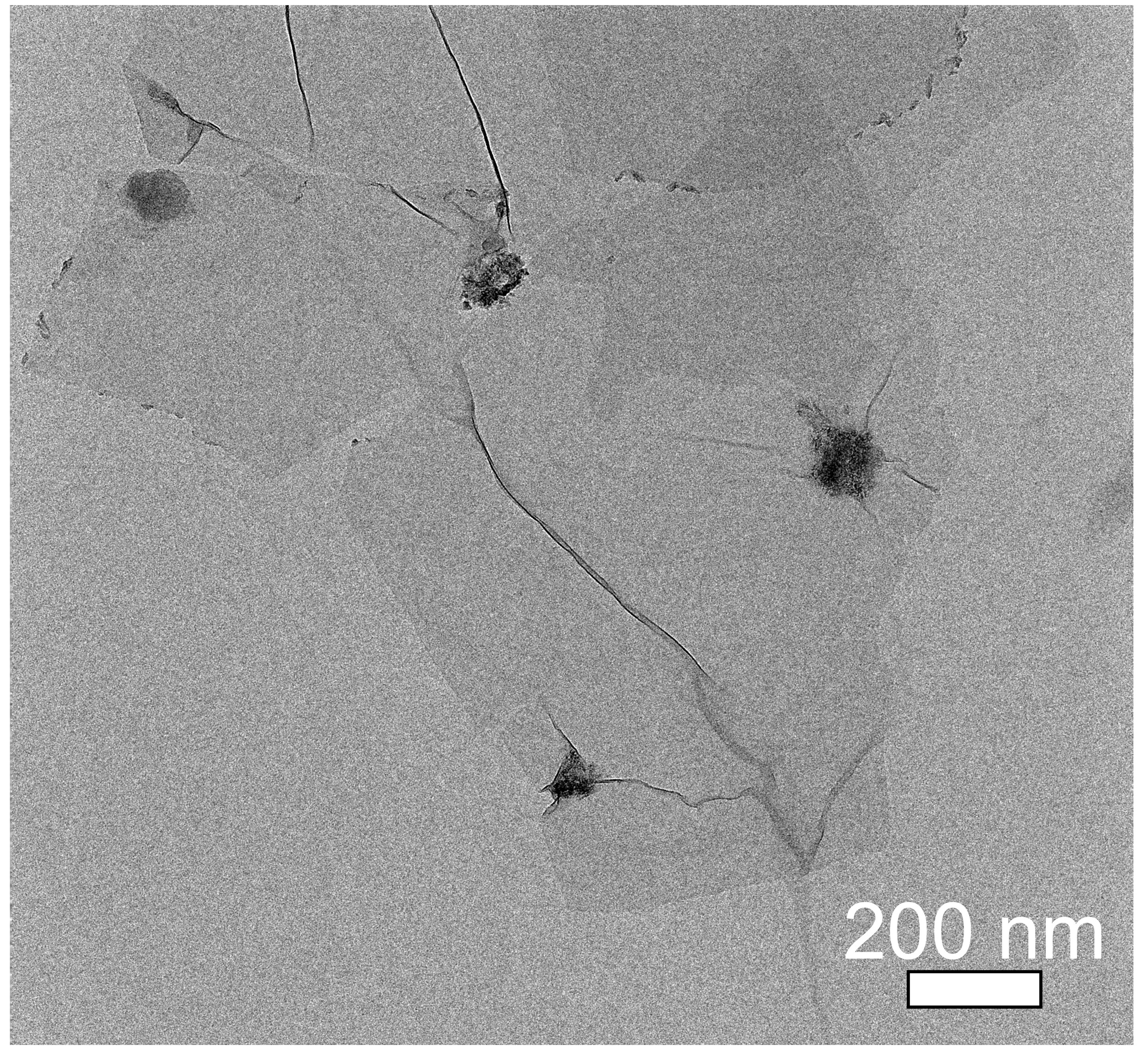


**Figure S13*.*** TEM image of S-MXene sealed and stored at 5°C for 180 days.


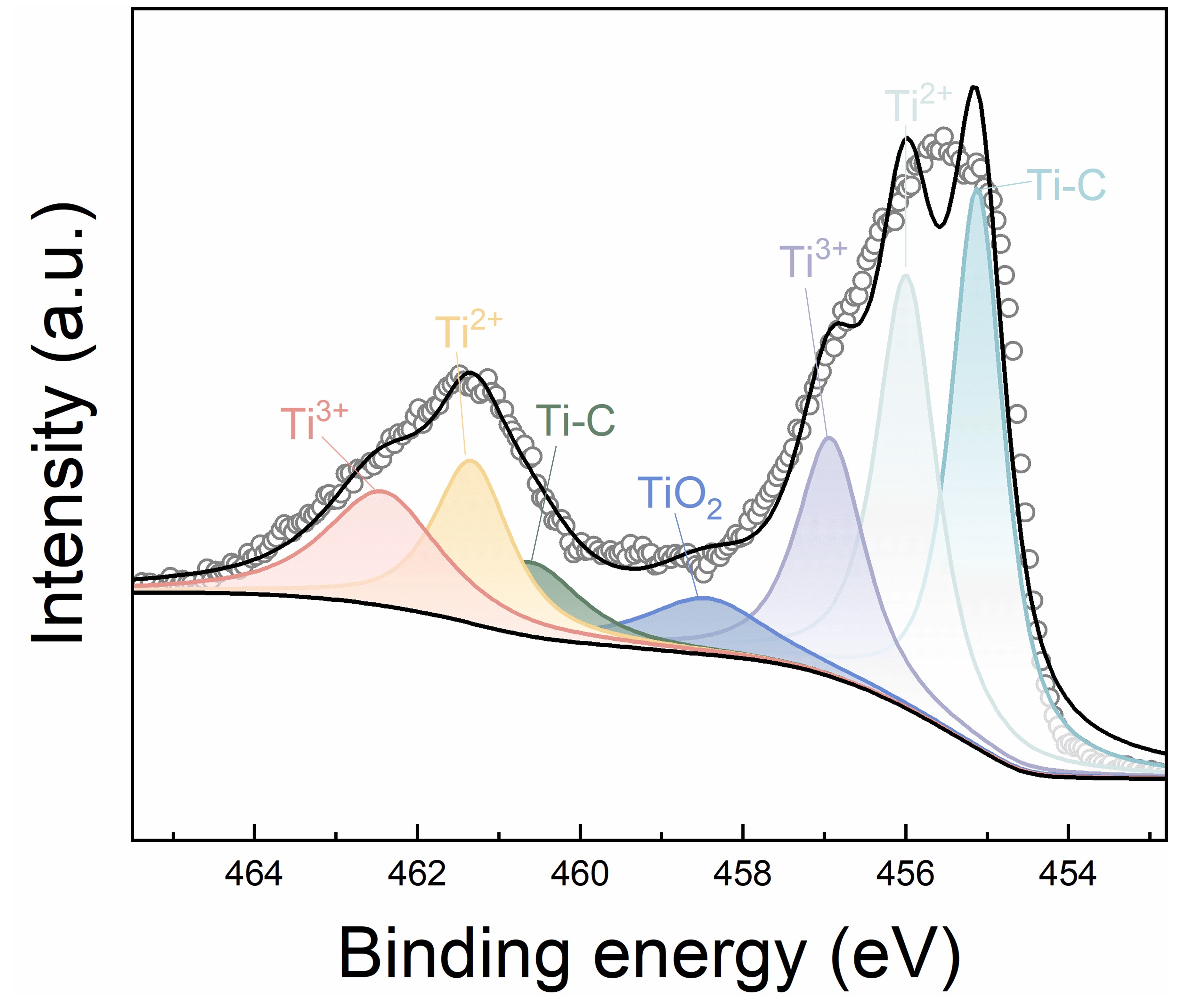


**Figure S14*.*** High-resolution XPS spectra of Ti 2p for S-MXene after being sealed and stored at 5°C for 180 days.


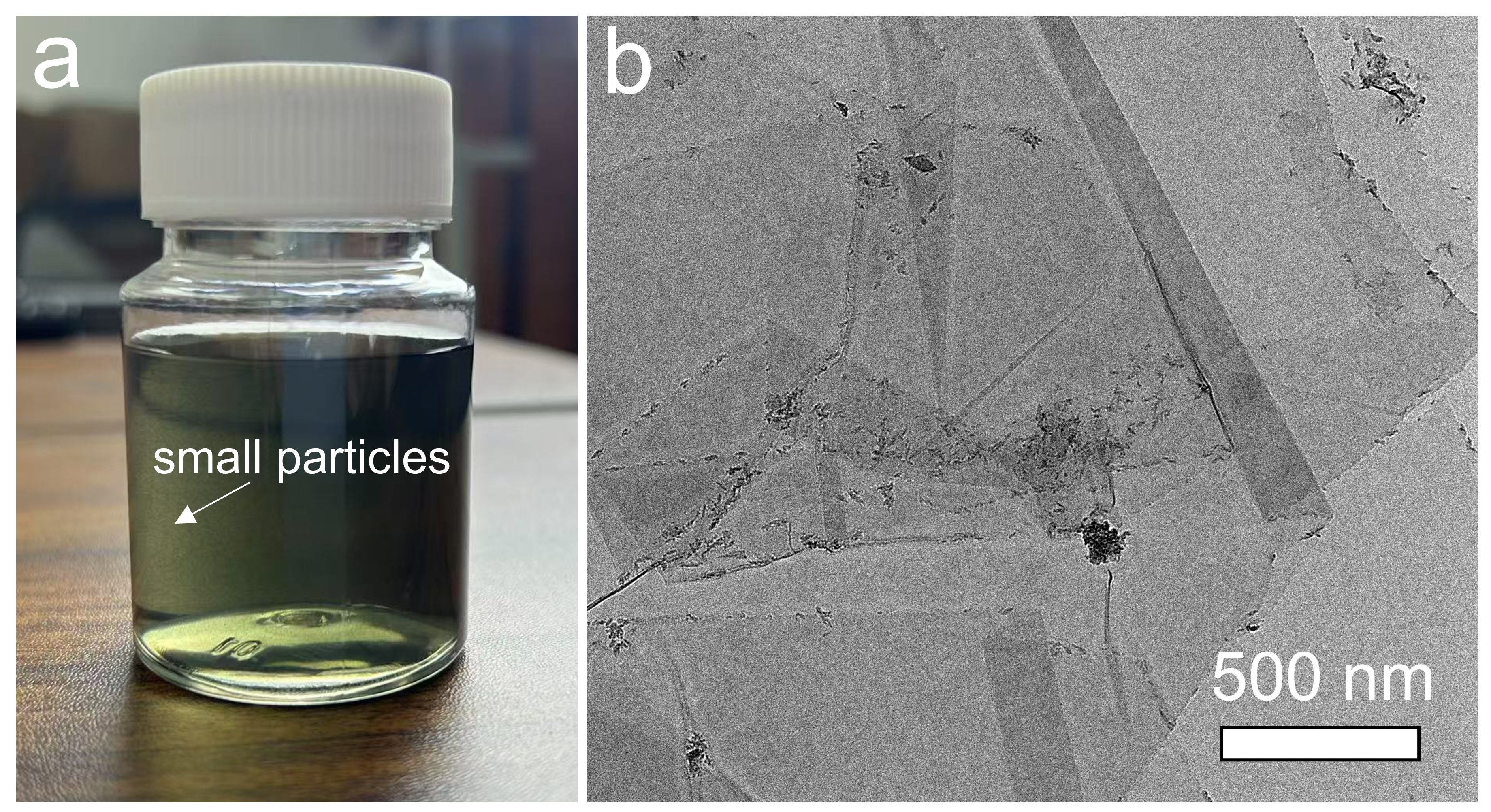


**Figure S15*.*** (a) Surface redispersibility of traditional semi-solid MXene sealed and stored at 5°C for 180 days. (b) TEM image of the internal region of traditional semi-solid MXene redispersed after being sealed and stored at 5°C for 180 days.


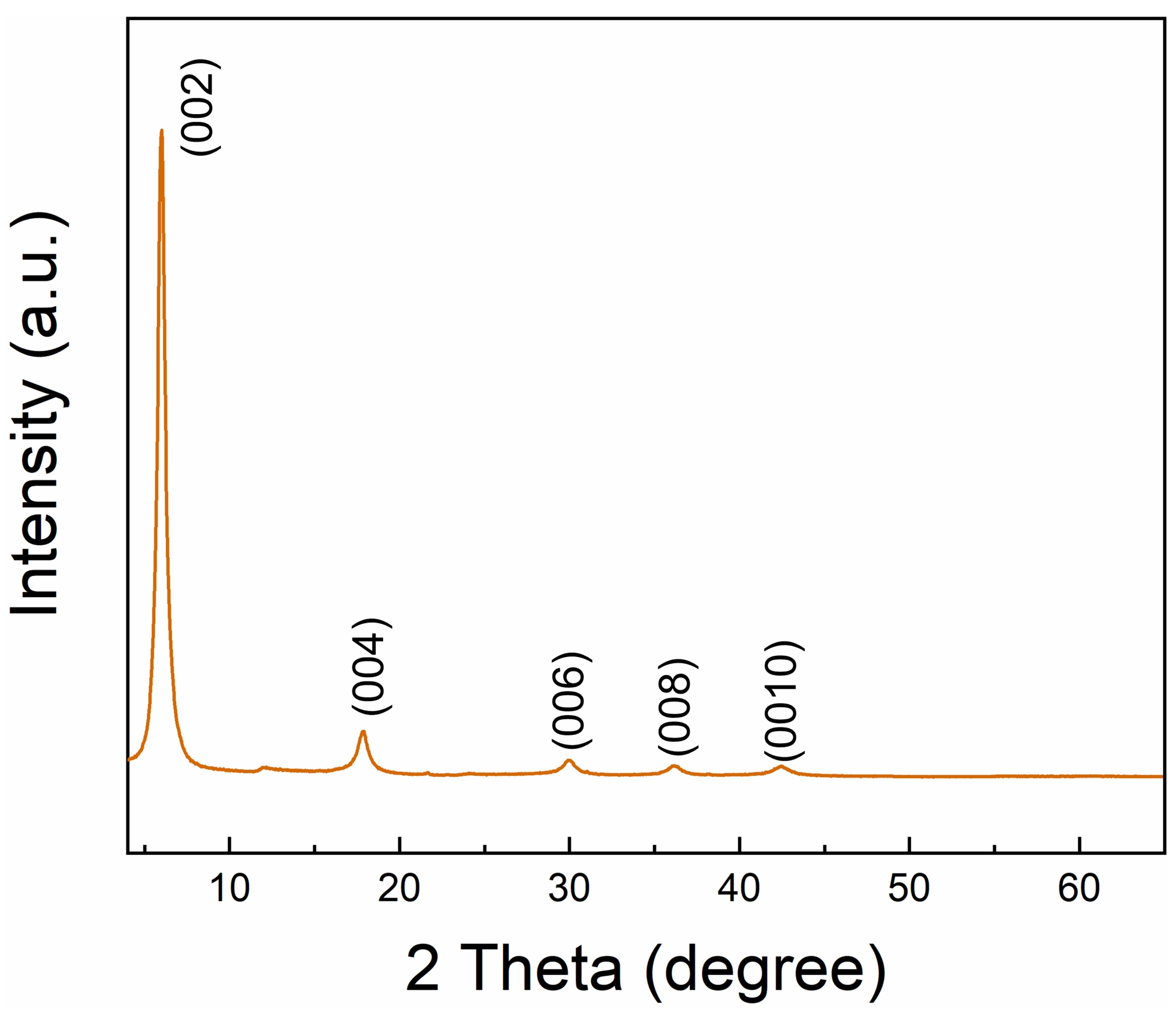


**Figure S16.** XRD spectrum of S-MXene after being sealed and stored at 5°C for 180 days.


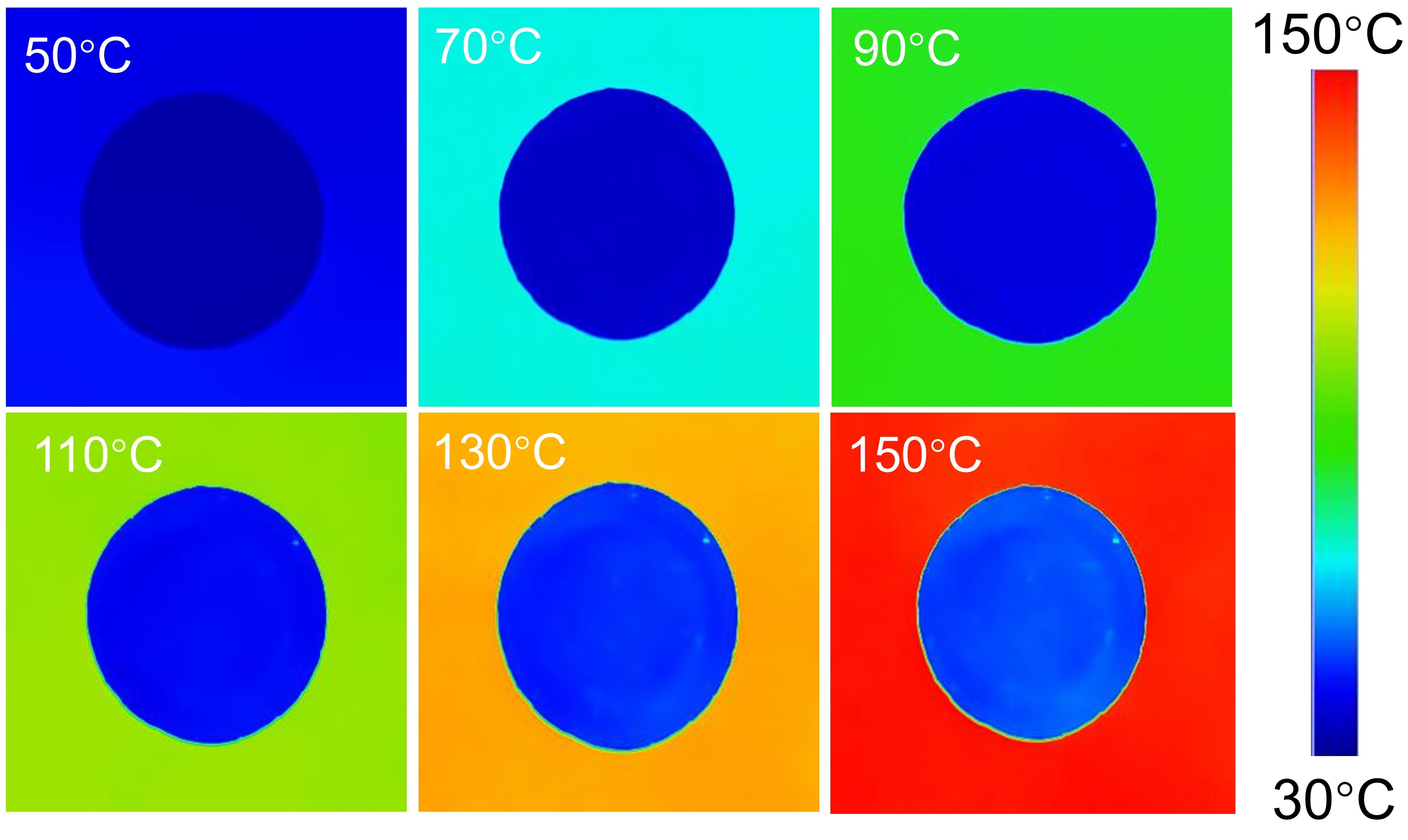


**Figure S17.** Infrared thermographic images of S-MXene film on a heating stage at different temperatures.


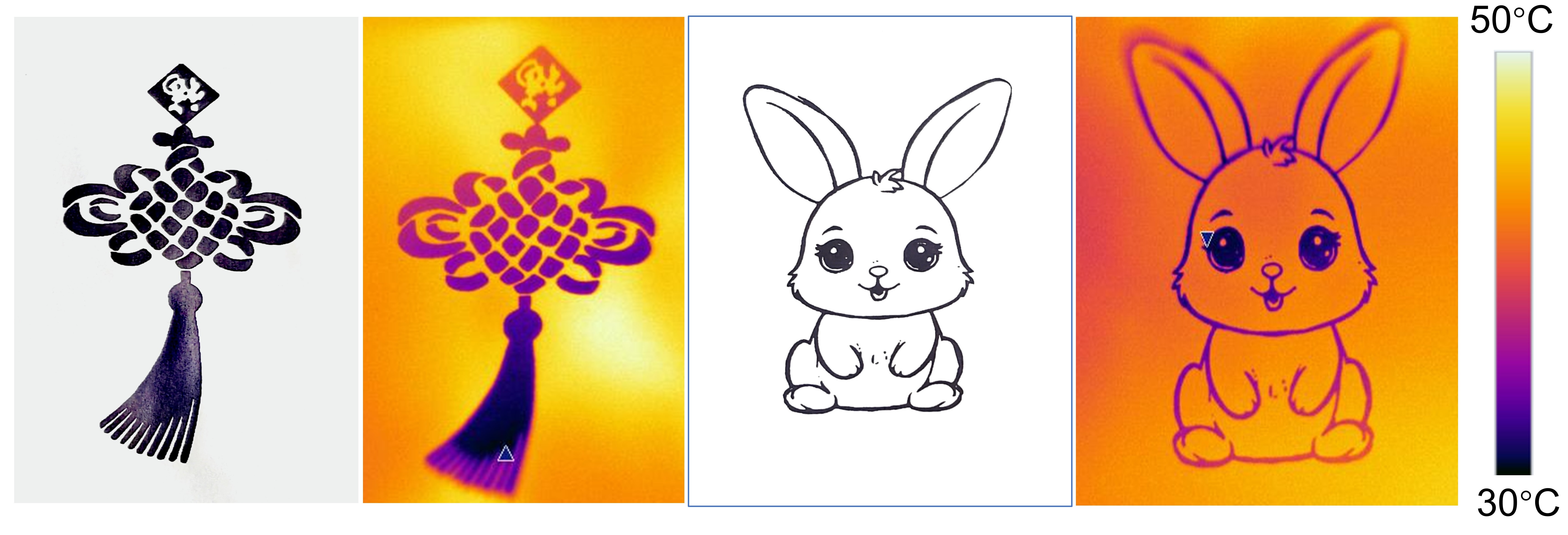


**Figure S18.** Various patterns of high-concentration slurry screen-printed from S-MXene conversion and their infrared thermographic images.


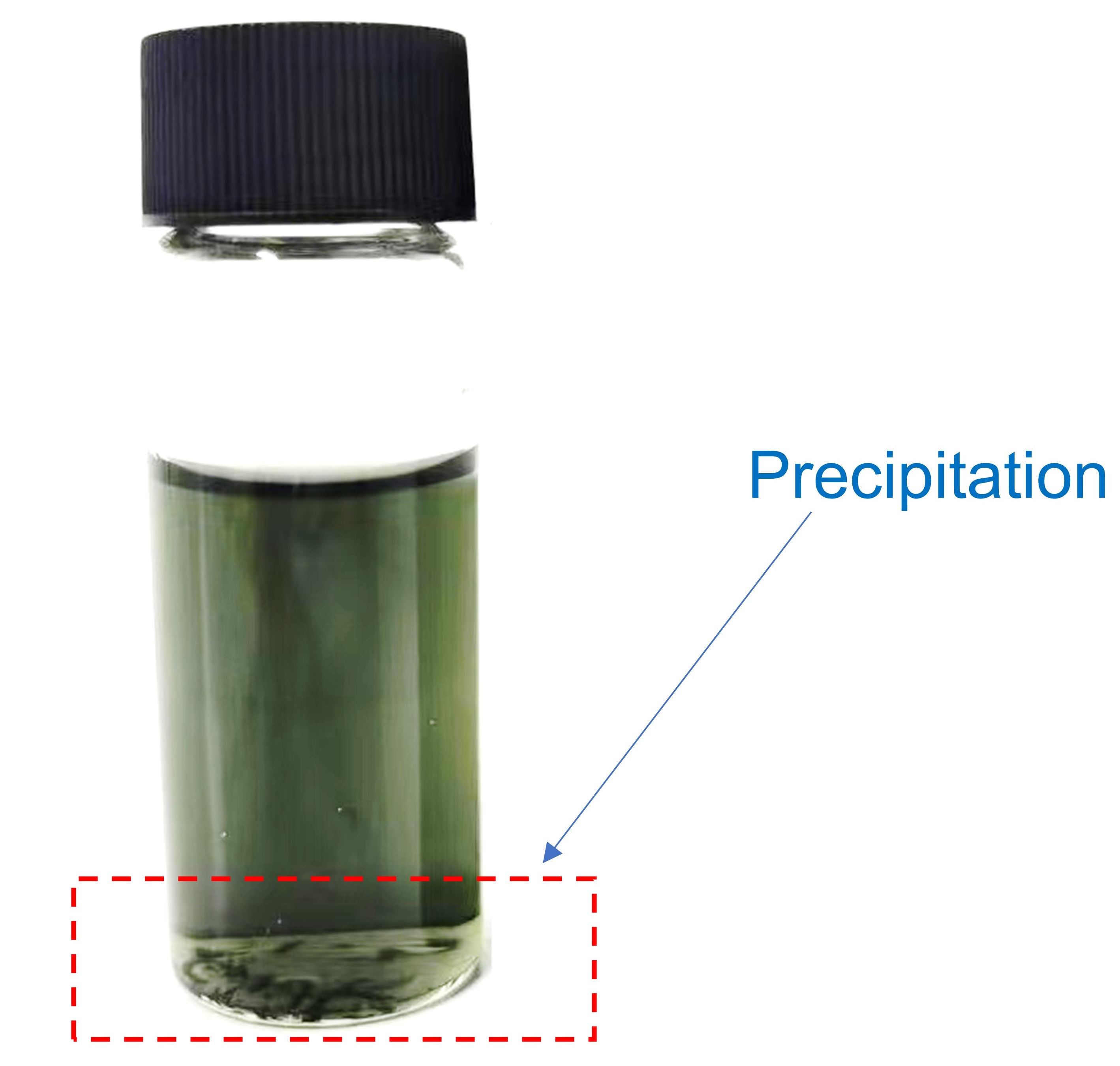


**Figure S19.** Redispersibility demonstration of the powder obtained from spray drying of MXene dispersion containing 1 wt% CNF.


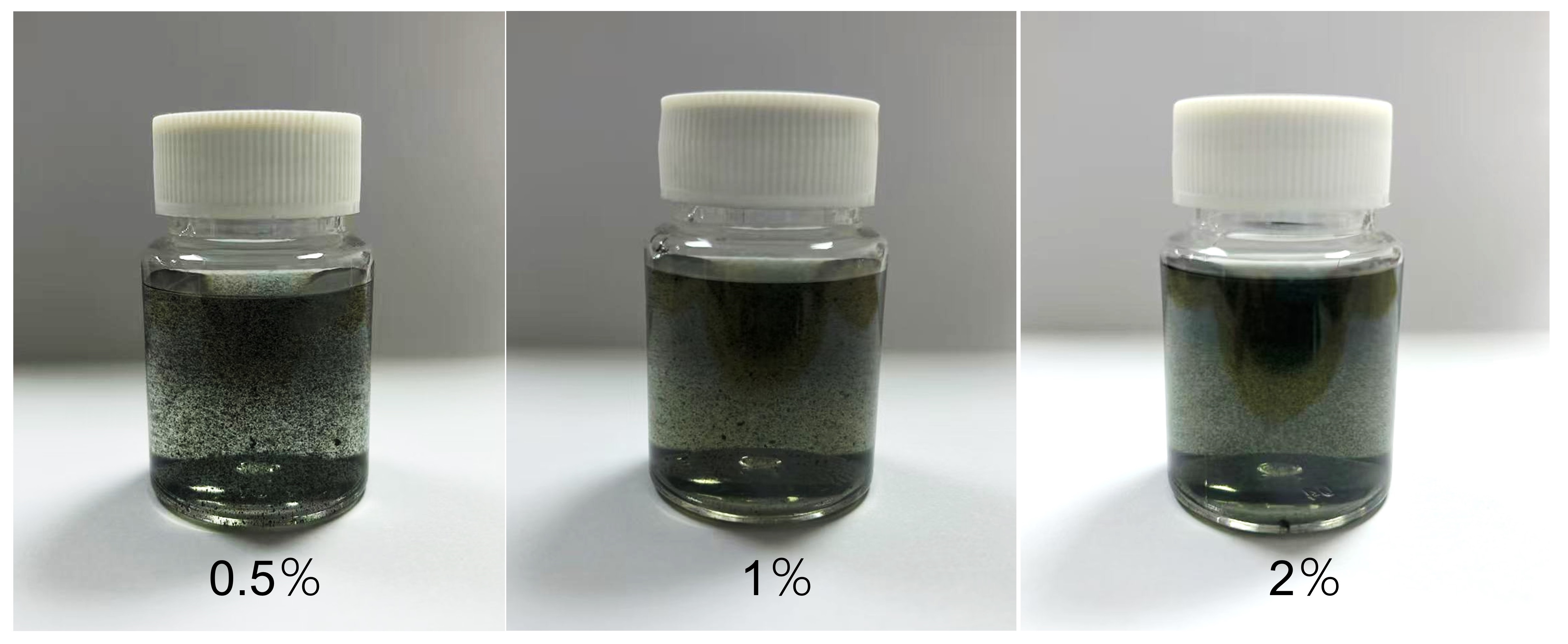


**Figure S20.** Redispersibility demonstration of the powder obtained from spray drying of MXene dispersion containing different amounts of LNC (0.5, 1, 2 wt%) beyond the time-dependent period.


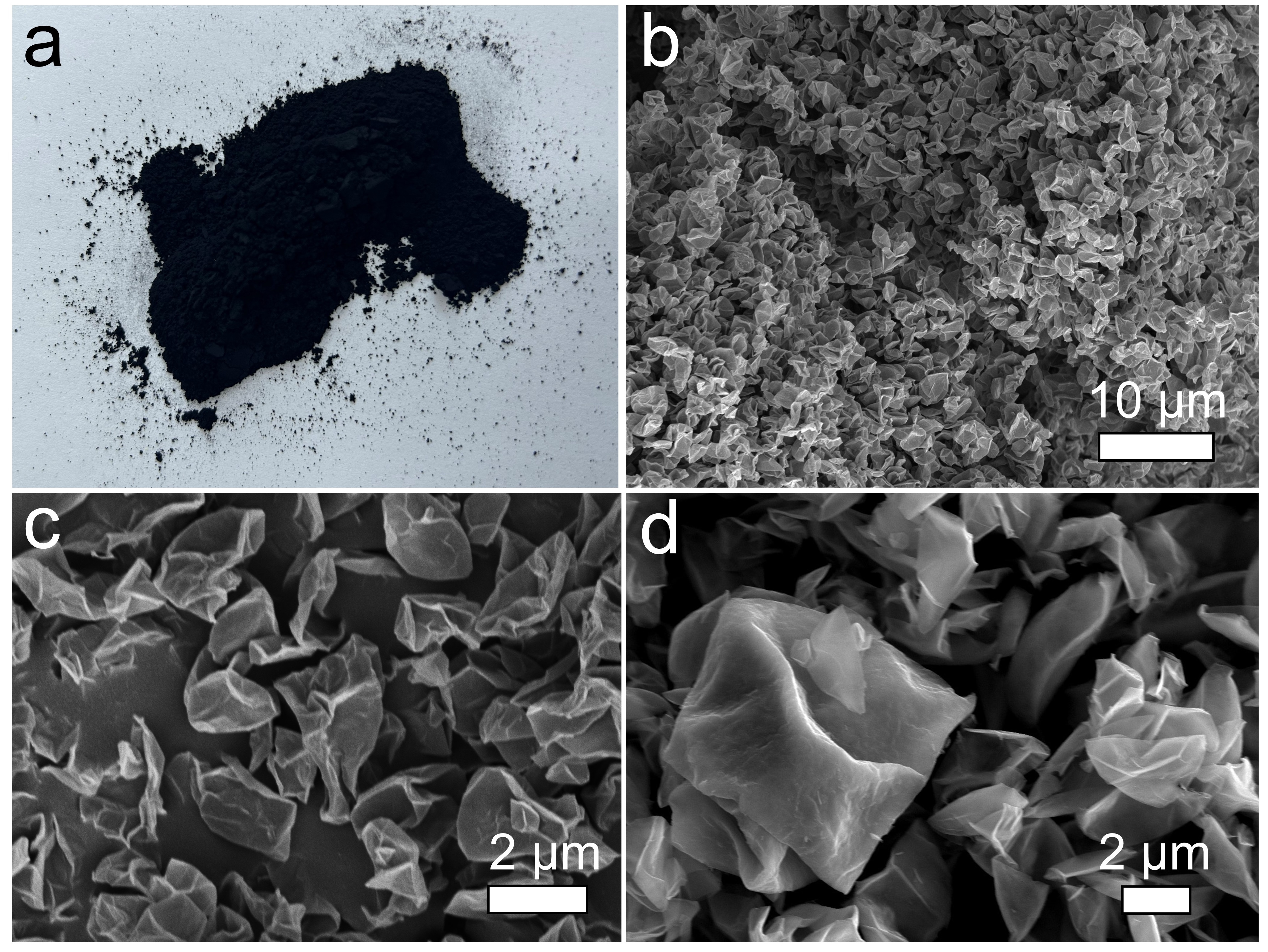


**Figure S21.** Photographs of (a) the powder obtained from spray drying of MXene dispersion containing 1 wt% LNC and (b-d) corresponding SEM images.


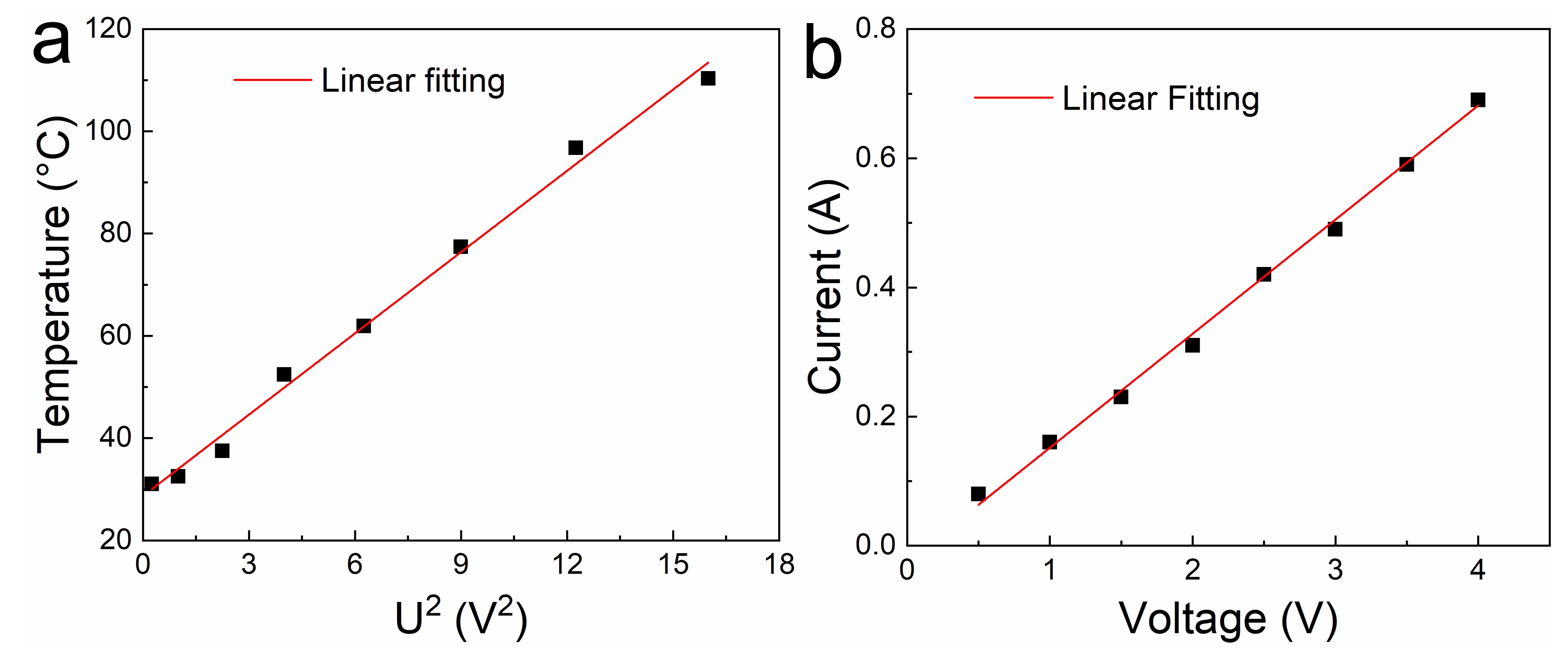


**Figure S22.** Conductive fabric: (a) relationship between saturation temperature and the square of the driving voltage, and (b) relationship between current and driving voltage.


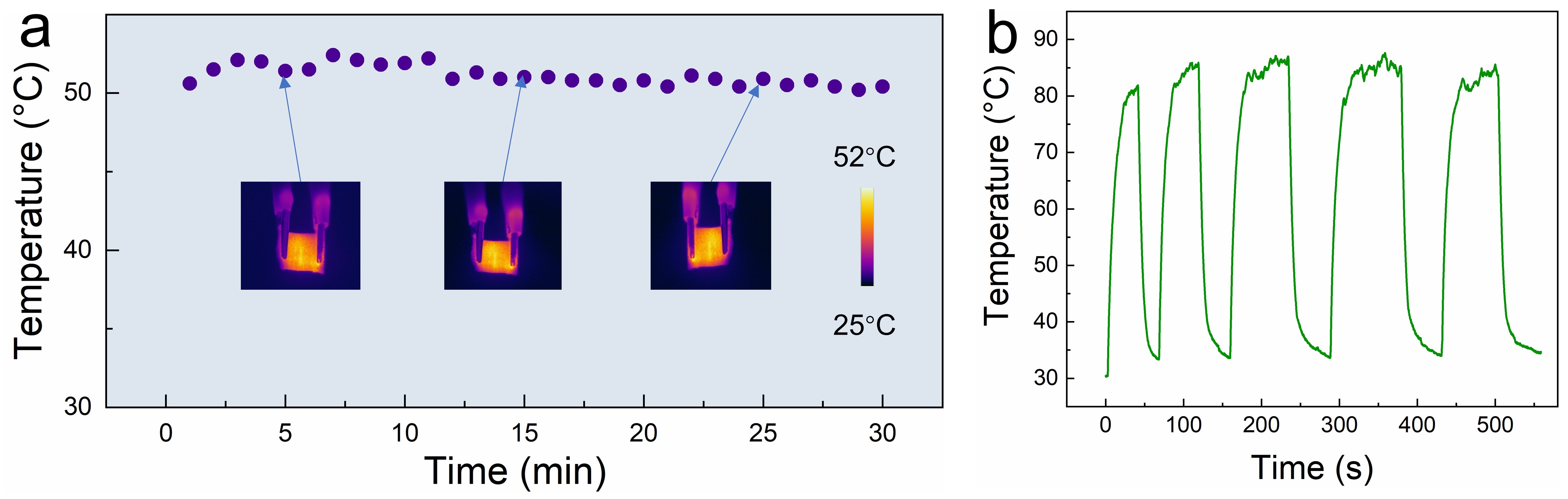


**Figure S23.** (a) Temperature change of conductive fabric under constant 2 V voltage for 30 min. (b) Temperature change of conductive fabric under cyclic heating/cooling.


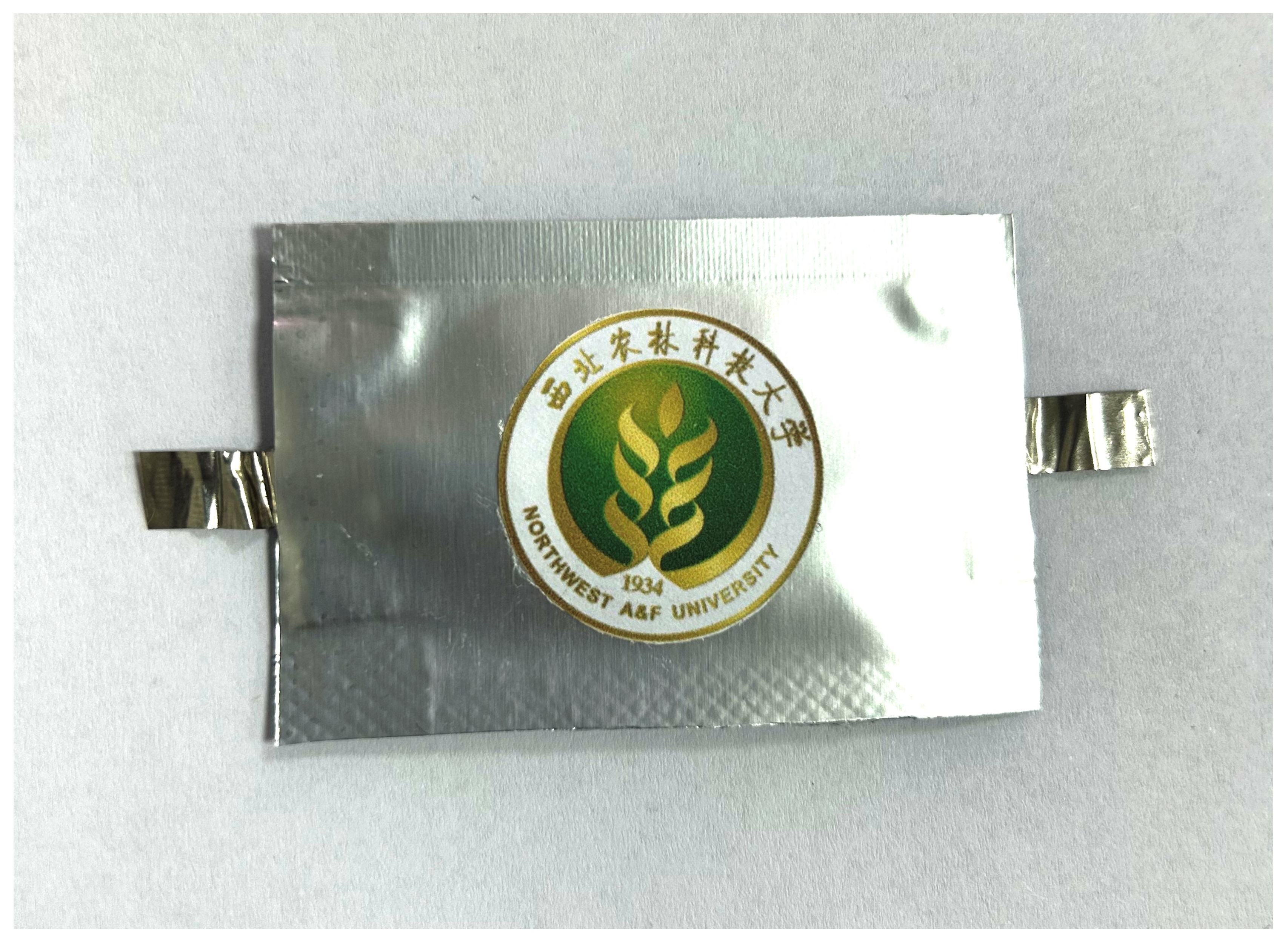


**Figure S24.** Photograph of the symmetric supercapacitor assembled with MXene powder as the active material.


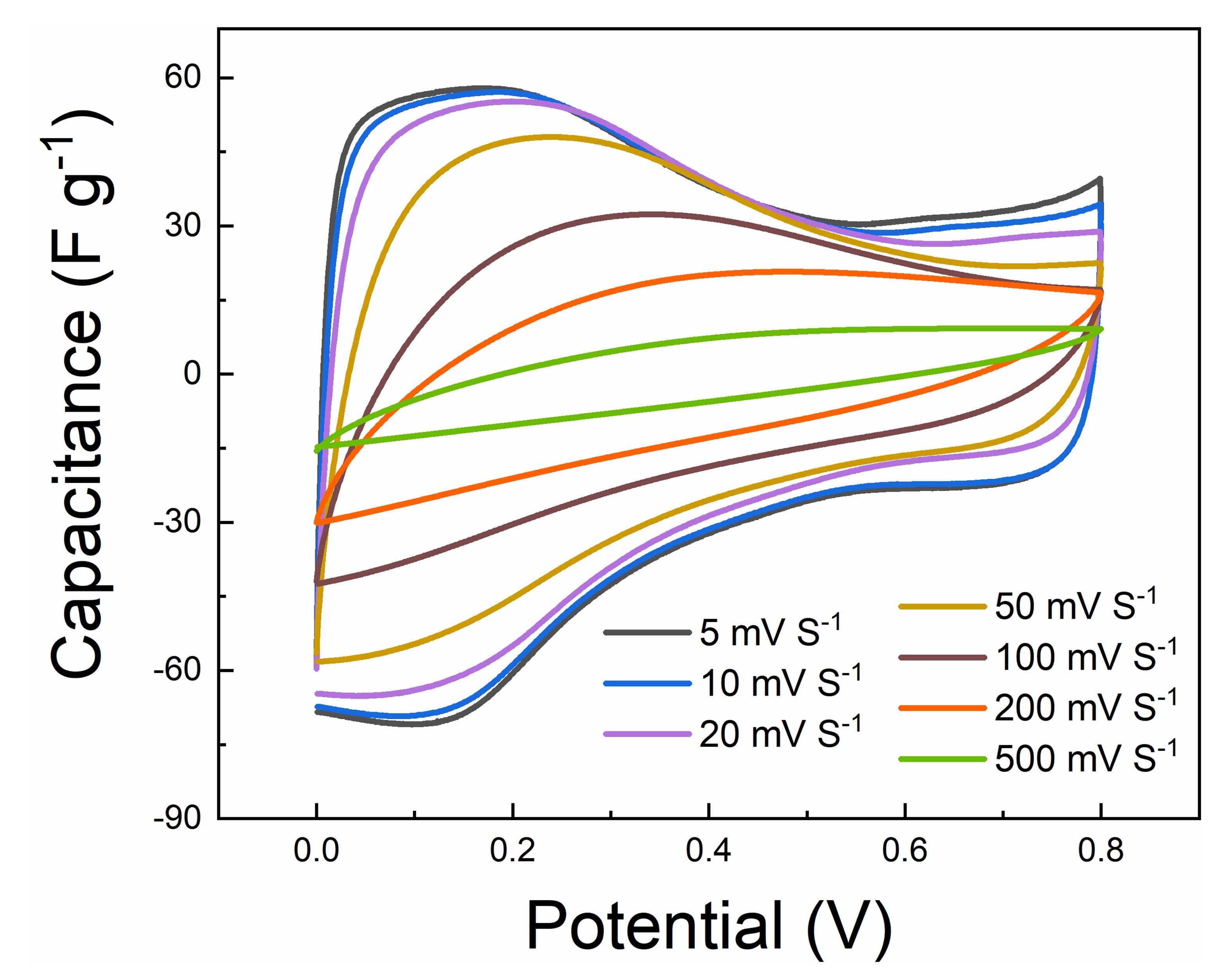


**Figure S25.** CV curves of MXene film-based symmetric supercapacitor.


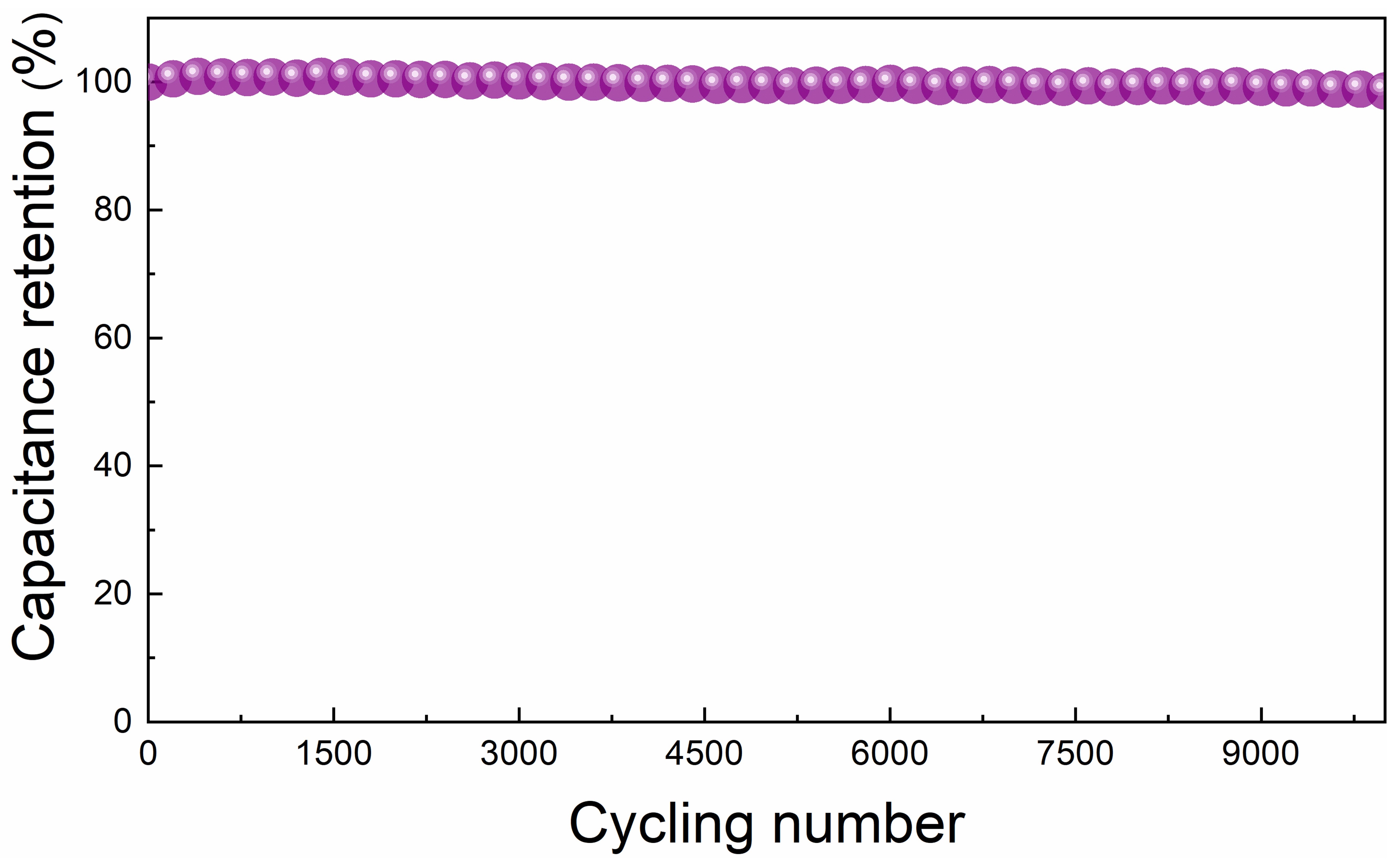


**Figure S26.** Capacitance retention of symmetrical supercapacitor assembled with structurally fixed MXene powder.


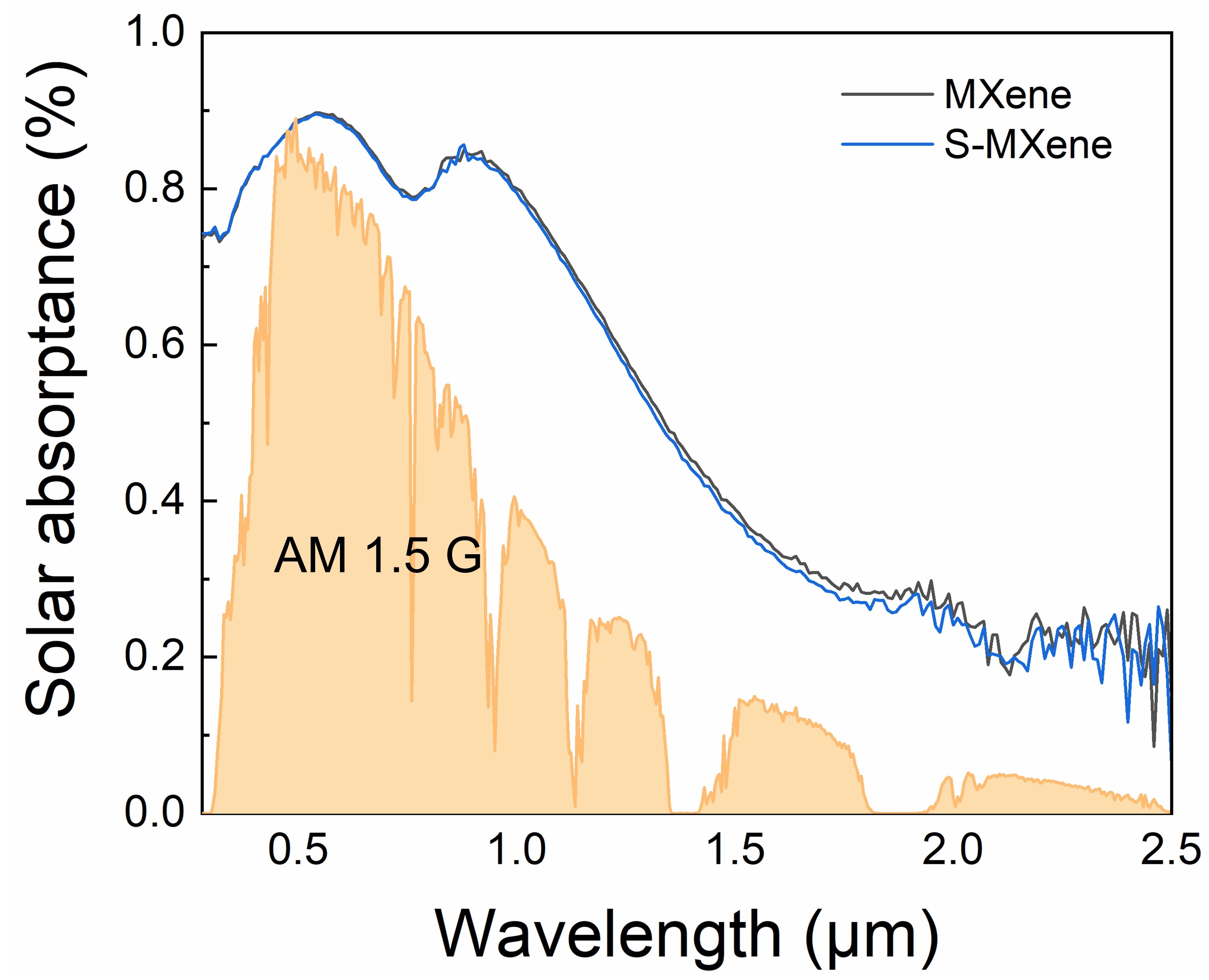


**Figure S27.** UV-vis-NIR absorption spectra of MXene and S-MXene.


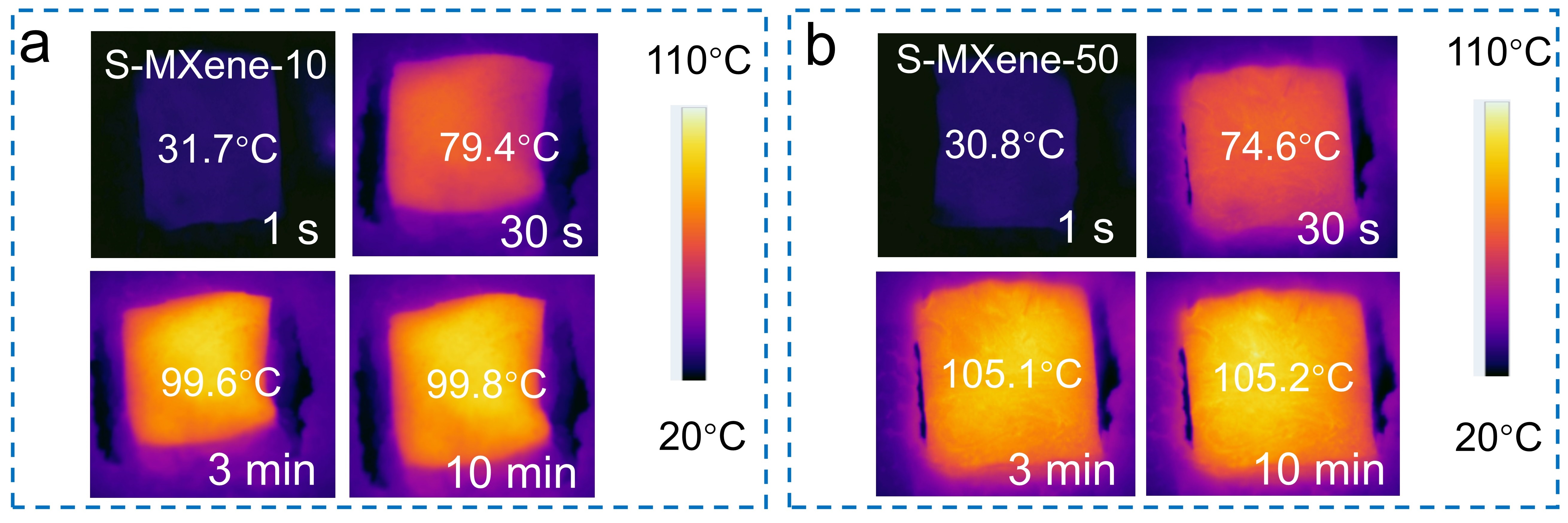


**Figure S28.** Infrared thermal images of (a) S-MXene-10 and (b) S-MXene-50 gels under one sunvirradiation.

**
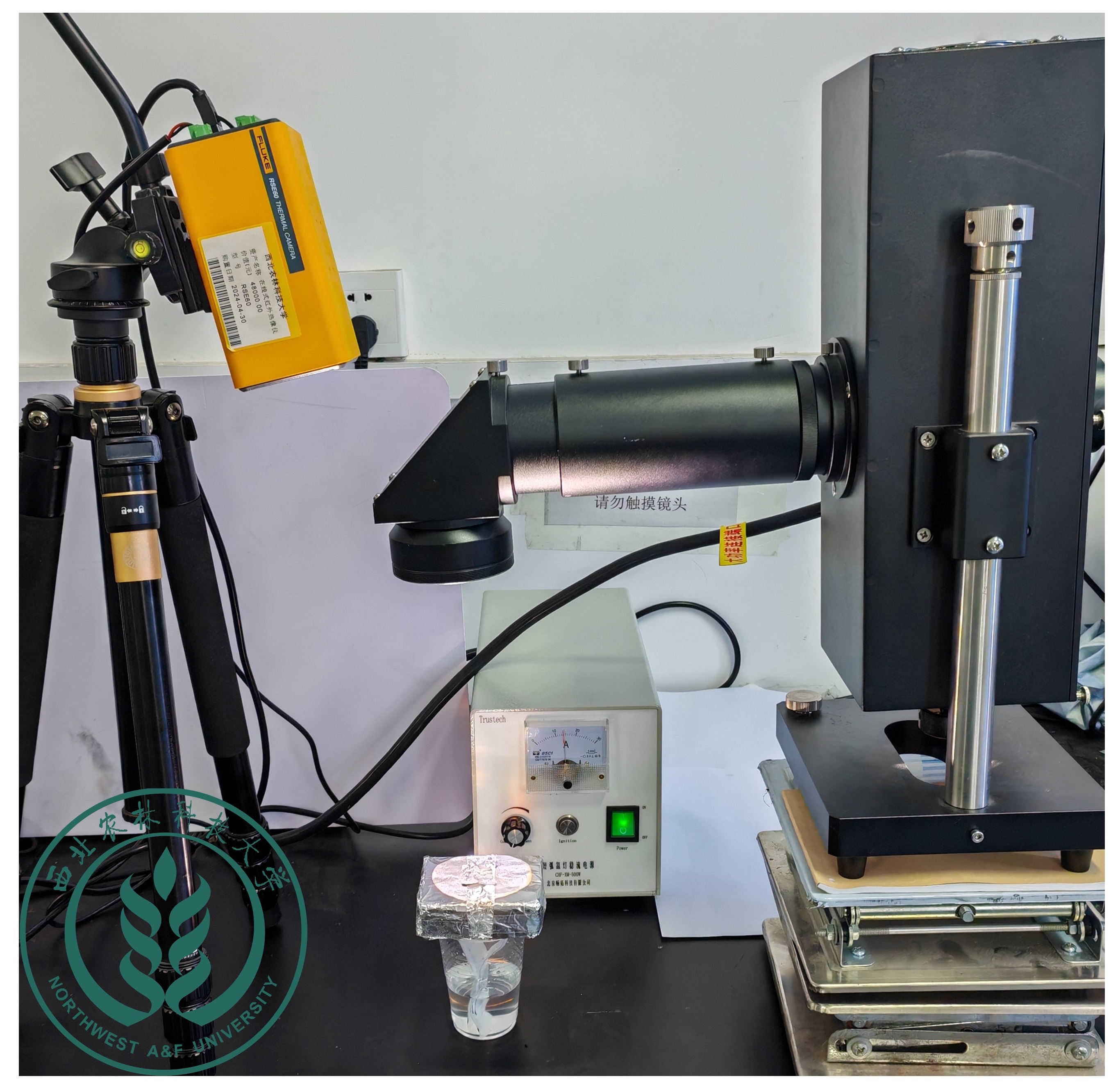
**

**Figure S29.** Photograph of the interface evaporation device using the S-MXene-30 gel sample under one sun irradiation.

**
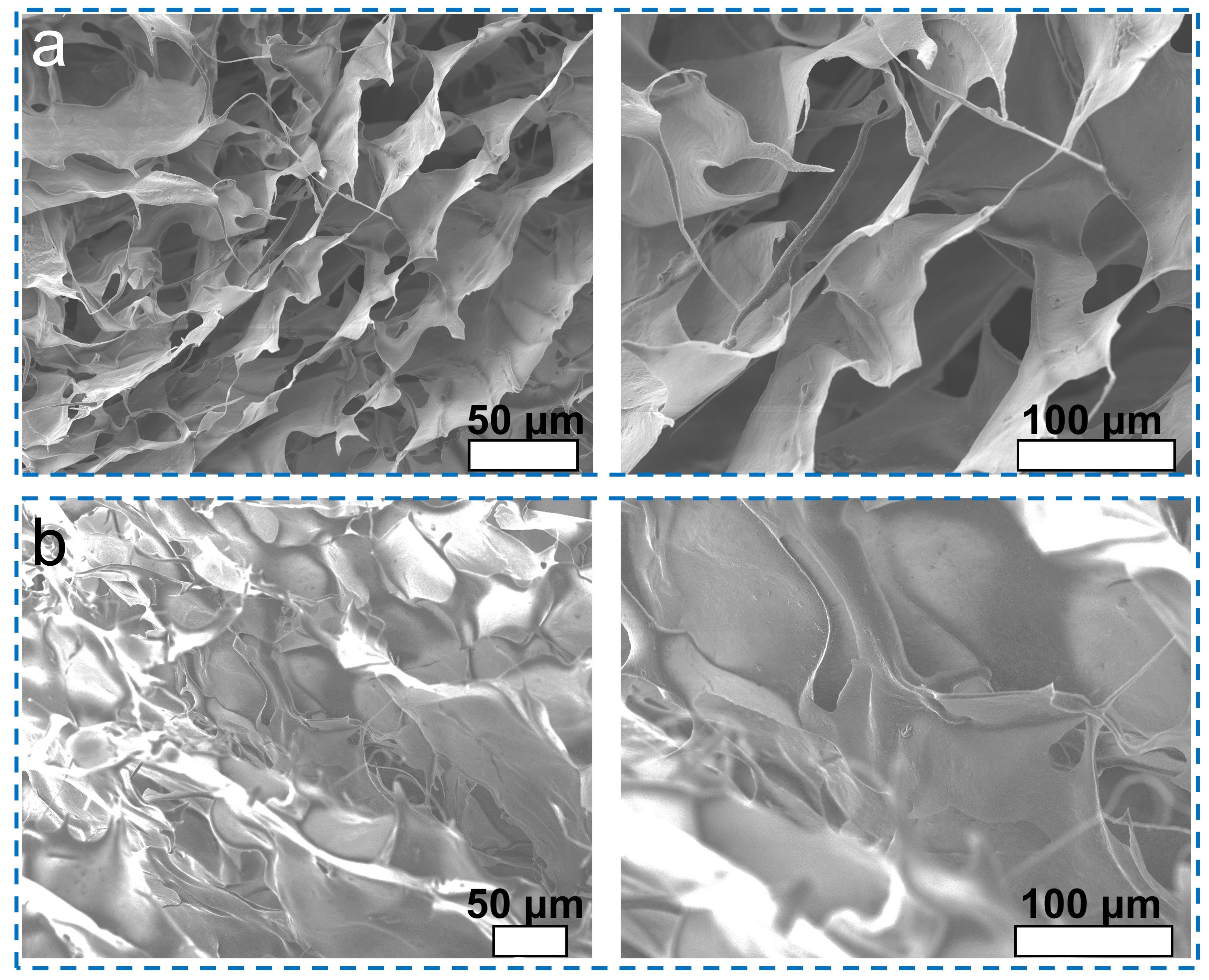
**

**Figure S30*.*** SEM images of (a) S-MXene-30 gel and (b) S-MXene-50 gel.


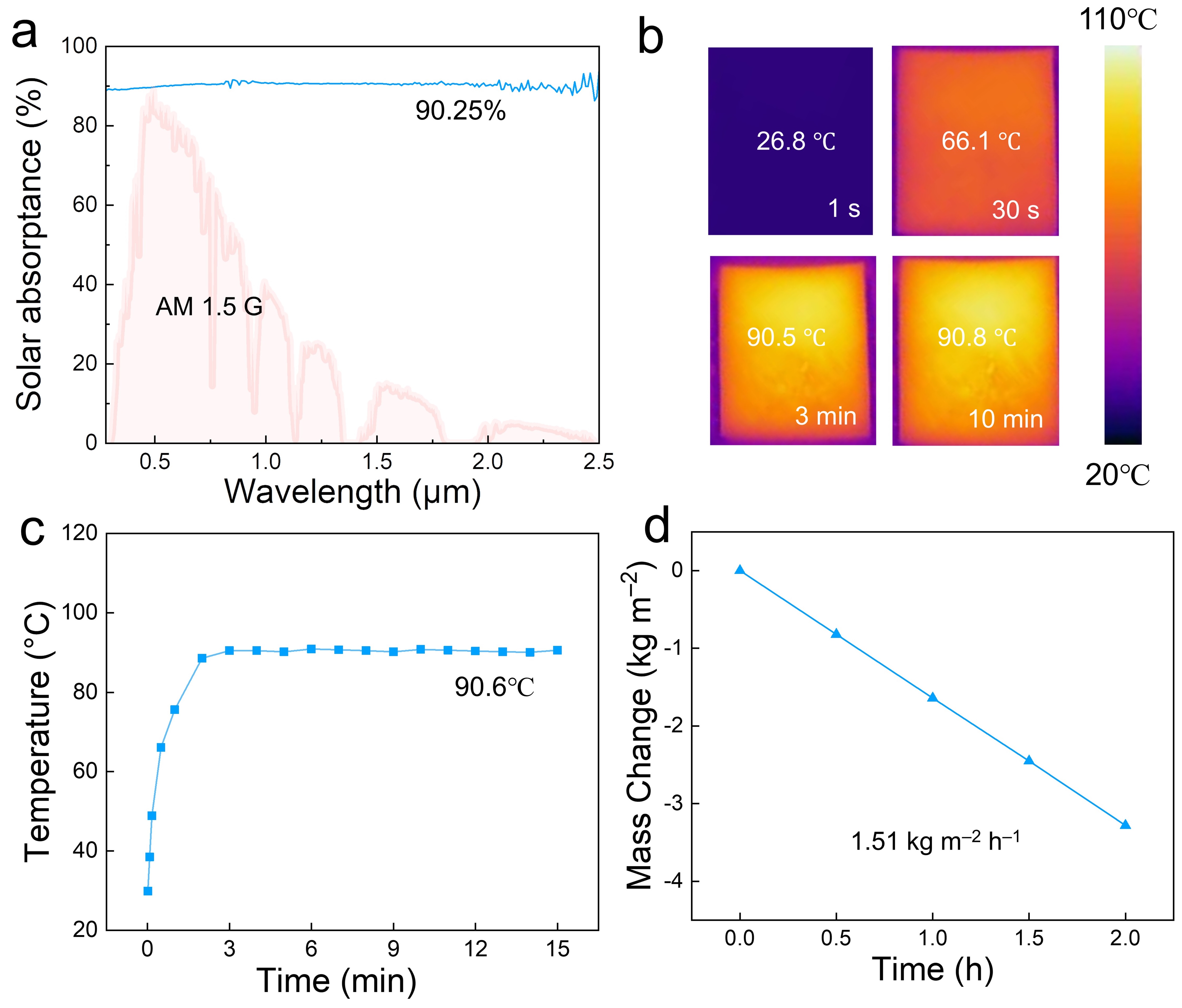


**Figure S31.** (a) UV-Vis-NIR absorption spectra of the CB-16 composite hydrogel (16 wt% carbon black incorporated in SA, particle size 10-15 nm). (b) Infrared thermal images of the dry CB-16 hydrogel under one-sun illumination. (c) Temperature rise of the dry CB-16 hydrogel over time under one-sun illumination. (d) Mass change of the CB-16 hydrogel over time under one-sun illumination.

Supporting Videos

**Video S1.** Molecular dynamics simulation depicting the shortest distance between the hydrogen atoms of the hydrophilic groups on the LNC segments and the hydrogen atoms on the MXene surface.

**Video S2.** Molecular dynamics simulation depicting the shortest distance between the hydrogen atoms of the hydrophobic groups and the hydrogen atoms on the MXene surface.

**Video S3.** Demonstration of three series-connected S-MXene-based symmetric supercapacitors stably powering an LED light for an extended period.
